# Transcriptomic profiling of a canine malignant eyelid melanoma

**DOI:** 10.29374/2527-2179.bjvm004025

**Published:** 2025-12-12

**Authors:** Wallax Augusto Silva Ferreira, Antonio Gomes Costa Neto de Sousa, Thamirys Aline Silva Faro, Edivaldo Herculano Correa de Oliveira

**Affiliations:** 1 Laboratory of Cytogenomics and Environmental Mutagenesis, Environment Section (SEAMB), Evandro Chagas Institute (IEC), Ananindeua, PA, Brazil.; 2 Institute of Exact and Natural Sciences, Faculty of Natural Sciences, Federal University of Pará (UFPA), Belém, PA, Brazil.

**Keywords:** eyelid melanoma, gene expression, comparative oncology, differential gene expression, chemokine pathway, melanoma palpebral, expressão gênica, oncologia comparada, expressão gênica diferencial, quimiocinas

## Abstract

Despite its aggressive behavior and translational potential, malignant eyelid melanoma in dogs remains under-researched in veterinary oncology. This study aimed to elucidate the molecular landscape of a malignant eyelid melanoma in a 4-year-old female dog of an undefined breed. Differential gene expression analysis revealed 1,437 significantly altered genes. Notably, the upregulated genes were enriched in immune-related pathways, such as chemokine signaling (*CCL3, CCL4, CCL8, CCL23, CCL24, CXCL8*, and *CXCL10*), whereas the downregulated genes were associated with cytoskeletal integrity processes and hormonal signaling. MicroRNA analysis highlighted *miR-134-5p* and *miR-146a-5p* as potential regulators of oncogenic pathways, underscoring their diagnostic and therapeutic importance. In addition, comparative analyses revealed conserved gene signatures in human basal cell carcinoma of the eyelids, emphasizing the importance of canine models in translational oncology. This comprehensive molecular characterization provides new insights into the pathogenesis of canine eyelid melanoma and identifies key biomarkers and pathways that may be relevant for future therapeutic interventions in veterinary and comparative oncology.

## Resumo

O melanoma palpebral maligno em cães ainda é pouco estudado na oncologia veterinária, apesar do seu comportamento agressivo e potencial translacional. Este estudo teve como objetivo elucidar o panorama molecular de um melanoma palpebral maligno em uma cadela de 4 anos, sem raça definida. A análise da expressão gênica diferencial revelou que 1.437 genes estão significativamente alterados. Os genes com expressão aumentada foram enriquecidos em vias relacionadas ao sistema imunológico, como a sinalização de quimiocinas (*CCL3*, *CCL4*, *CCL8*, *CCL23*, *CCL24*, *CXCL8* e *CXCL10*), enquanto os genes com a expressão reduzida foram associados a processos de integridade do citoesqueleto e sinalização hormonal. Em relação aos miRNAs, identificamos os *miR-134-5p* e *miR-146a-5p* como potenciais reguladores de vias oncogênicas, ressaltando sua importância diagnóstica e terapêutica. Além disso, as análises comparativas revelaram assinaturas genéticas conservadas com o carcinoma basocelular palpebral humano, enfatizando a relevância de modelos caninos para a oncologia translacional. Esta caracterização molecular abrangente fornece novos *insights* sobre a patogênese do melanoma palpebral canino e identifica biomarcadores e vias chaves que podem ser relevantes para futuras intervenções terapêuticas na oncologia veterinária e comparada.

## Introduction

Canine ocular melanomas are a heterogeneous group of primary tumors arising in the eyelids, conjunctiva, and uveal tract (Wang & Kern, 2015). Their biological behavior ranges from benign melanocytomas to highly invasive malignant melanomas, with prognosis largely determined by the location and cytological features (Giuliano et al., 1999). For example, uveal melanomas often cause secondary complications, such as glaucoma and hyphema, while limbal melanomas generally progress more slowly and have more favorable clinical outcomes (Diters et al., 1983). Prognostic factors for ocular melanomas include tumor type, cytological features, and the presence of metastases, although metastasis is less common in canine ocular melanomas than in other species (Ryan & Diters, 1984). Recent advances in diagnostic imaging and refined histopathological classifications have improved disease characterization and therapeutic strategies in veterinary oncology (Alshammari et al., 2025; Avallone et al., 2021; Cassali et al., 2001).

Eyelid neoplasms are the most common periocular tumors in dogs (Wang et al., 2019). They typically occur in older animals (average onset: 8 years) and show breed-specific susceptibility but no apparent sex-specific predisposition (Camargo et al., 2008; Tuntivanich et al., 2004). Although most cases are benign (Tuntivanich et al., 2004; Wang & Kern, 2015), approximately 8% exhibit malignant features that are rarely observed in younger dogs (Riis et al., 2002). Malignant eyelid melanomas are characterized by dark-pigmented nodular masses and an aggressive clinical course, often accompanied by pulmonary or hepatic metastases (Roberts et al., 1986). Because of their proximity to critical anatomical structures, including the eyeballs, brain, and paranasal sinuses, these tumors can cause ocular discomfort, visual impairment, inflammatory complications such as uveitis and glaucoma, as well as significant cosmetic disfigurement (Finn et al., 2008; Luz et al., 2023; Wang & Kern, 2015). Therefore, early detection, followed by surgical excision with clear margins, potentially combined with cryotherapy to optimize the cosmetic outcome, is crucial for achieving a favorable prognosis (Han & Kim, 2022).

High-throughput genomic and transcriptomic technologies have transformed cancer biology by revealing the molecular signatures and pathways that are central to tumorigenesis (Ferreira & Oliveira, 2022; Ferreira et al., 2022). In canine oncology, most studies have focused on cutaneous and oral melanomas (Gillard et al., 2014; Poorman et al., 2015), whereas malignant eyelid tumors remain poorly characterized. Given the remarkable biological and molecular similarities between canine and human melanomas, dogs are valuable translational model (Gillard et al., 2014; Rahman et al., 2020; Simpson et al., 2014). Comparative analyses not only advance the mechanistic understanding of canine melanoma but also highlight conserved oncogenic pathways relevant to human disease (Simpson et al., 2014).

This study aimed to elucidate the molecular landscape of canine malignant eyelid melanoma using an integrative transcriptomics approach.

## Material and methods

### Tissue samples

A 4-year-old female dog of an undefined breed underwent surgical excision of a tumor on the upper eyelid, along with adjacent healthy tissue, at the Veterinary Hospital of the Federal Rural University of Amazônia (HOVET-UFRA, Castanhal, Pará, Brazil). Part of the tissue samples were frozen and stored at -80°C for subsequent RNA extraction. The remaining tissues were fixed in formalin and stained with hematoxylin and eosin (H&E). Histopathological examination confirmed that the lesion was malignant eyelid melanoma.

### RNA isolation

RNA was isolated from frozen dog tissues in RNAlater (Invitrogen™, Carlsbad, CA, USA, Cat# AM7020) using the ReliaPrep™ RNA Miniprep System Kit (Promega™, Madison, WI, USA, Cat# Z6112). The quality and quantity of total RNA were determined using Nanodrop 2000 (Thermo Fisher Scientific™, Wilmington, DE, USA, Cat# ND-2000,) and Agilent 2200 Tapestation with High Sensitivity RNA ScreenTape (Agilent, Santa Clara, CA, USA). All samples forwarded for cRNA amplification and labeling had an RNA integrity number (RIN) of >8 and total RNA amount of 100 ng.

### Microarray and gene expression analysis

Total RNA (100 ng) was reverse transcribed into double-stranded cDNA and labeled with cyanine 3 (Cy3) dye using a Low Input Quick Amp Labeling Kit, single color (Agilent, Cat# 5190-2305), according to the manufacturer’s instructions. Next, 600 ng of Cy3-labeled cRNA was fragmented and hybridized to a Canine (V2) Gene Expression Microarray, 4x44K (Agilent, Cat# G2519F-021193). Hybridized arrays were scanned using an Agilent scanner (Cat# G2565CA).

The intensity values of the scanned features were extracted using Feature Extraction software (version 10.7.3.1, Agilent), and raw data were processed using GeneSpring GX 14.5 software (Agilent, Cat# G3784AA) after quality control and robust multiarray average (RMA) quantile normalization (Ferreira et al., 2021). Quality control was performed using diagnostic plots including principal component analysis (PCA), boxplots, Pearson correlation, and MvA plots. All experiments were performed in triplicate.

The differentially expressed genes (DEGs) were identified using the paired Mann–Whitney test, with a Benjamini–Hochberg corrected p-value threshold of < 0.05 and an absolute fold-change (FC) of ≥3 (Ferreira et al., 2021). Functional enrichment analysis was performed using the ShinyGO v0.81 tool (Ge et al., 2020), and canine genes were mapped to human orthologs using the DAVID gene ID conversion tool (Sherman et al., 2022) and validated by the National Center for Biotechnology Information (NCBI) (https://www.ncbi.nlm.nih.gov/gene/).

To validate the DEGs identified in canine eyelid melanoma, we analyzed the GSE103439 dataset (Yunoki et al., 2018), which included normal human epidermal keratinocyte and basal cell carcinoma samples. It was selected as the reference dataset because of the lack of publicly available transcriptomic data on human eyelid melanomas.

### DEGs-associated microRNAs

To predict DEG-associated microRNAs (miRNAs), we integrated three main resources: (i) the DIANA-TarBase v.8.0 tool (Karagkouni et al., 2018), (ii) miRDB database (Chen & Wang, 2020), and (iii) miRWalk 3.0 database (Dweep et al., 2014). To ensure consistency, all candidate miRNAs were cross-validated across these platforms. To contextualize their functional implications, we conducted a KEGG pathway enrichment analysis using DIANA-miRPath v4.0 (Tastsoglou et al., 2023), a server specifically designed for target-based miRNA functional analysis. Analyses were performed with stringent parameters: (i) Targets: TarBase v8.0; (ii) TarBase targets: direct; (iii) Species: *Homo sapiens*; (iv) miRNA annotation: miRBase-v22.1; (v) Pathways: KEGG; (vi) Merge method: gene union; (vii) test method: classic analysis; (viii) P-value threshold: 0.05, with FDR correction. Pathway visualizations were generated using the Pathview package (Luo et al., 2017), with target genes highlighted by the number of regulating miRNAs (yellow: 1 miRNA, orange: 2 miRNAs, red: ≥ 3 miRNAs).

We also assessed the diagnostic efficiency of *hsa-miR-134-5p* and *hsa-miR-146a-5p* across The Cancer Genome Atlas (TCGA) Pan-Cancer cohorts. Receiver operating characteristic (ROC) curve analyses were performed using the CancerMIRNome database (Li et al., 2022) under default conditions, allowing independent assessment of each miRNA.

### Cancer hallmarks analysis

To determine which cancer hallmarks were associated with DEGs in canine eyelid melanoma, we utilized the Cancerhallmarks database (https://cancerhallmarks.com/) (Menyhart et al., 2025). This comprehensive resource contains over 6,763 hallmark-associated genes annotated using multiple mapping sources. This database enhances the utility of the hallmark concept by categorizing genes based on their biological functions. To assess the enrichment, distribution-based visualization was used to identify whether a gene was significantly represented in a hallmark category. The red line represents the threshold of statistical significance at adjusted p < 0.05. The hallmark enrichment plot evaluates the significance of the comparison between the studied and reference genes by displaying colored intersections.

### Cell type–specific enrichment analysis of genes

Next, we conducted a cell type–specific enrichment analysis (CSEA) using WebCSEA (Dai et al., 2022). This online tool employs the deTS algorithm (Dai et al., 2021) to calculate the raw *P*-value across 1,355 tissue and cell types. To address the potential bias arising from the varying lengths of signature genes and input gene lists, WebCSEA uses a permutation-based method to adjust raw p-values. This adjustment was based on rankings from over 20,000 gene lists derived from GWAS studies and a curated association of rare variants linked to human traits and diseases.

In our analysis, we considered target genes with a permutation-adjusted P < 0.001 as suggestive and a permutation-adjusted P < 3.7 × 10^-5^ (equivalent to 0.05/1355 tissue cell types) as experimentally significant. As suggested by the WebCSEA authors, the threshold for experimental significance may be too stringent; therefore, we report both raw and corrected p-values.

## Results and discussion

### Global gene expression analysis

**Figure 1:**
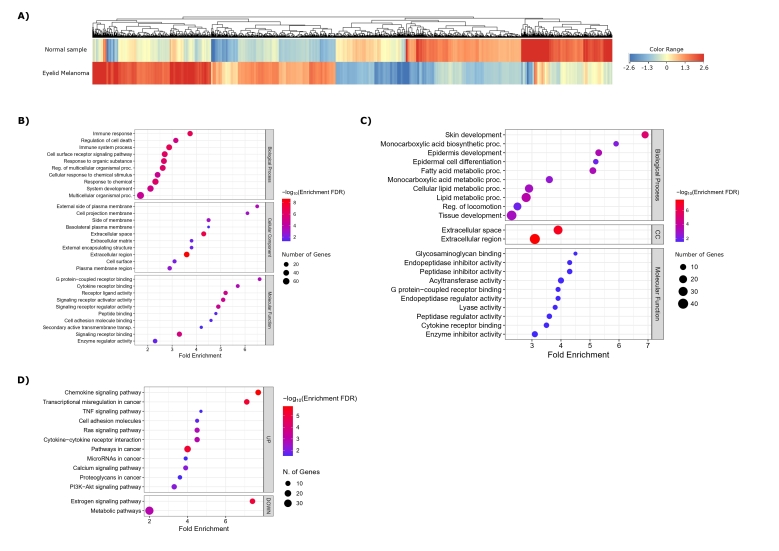
Functional analysis of differentially expressed genes (DEGs) in canine eyelid melanoma. **(A)** Hierarchical clustering of DEGs between canine eyelid melanoma (bottom row) and adjacent normal tissue (top row), with expression levels represented by a blue-to-red color scale indicating low to high expression, respectively. Clustering was performed using the Euclidean distance metric and Ward’s linkage method. **(B)** Gene Ontology (GO) enrichment analysis of upregulated and **(C)** downregulated DEGs from canine eyelid melanoma. The y-axis indicates the significantly enriched GO terms, while the x-axis indicates the fold enrichment. The dot size indicates the number of genes associated with each GO term, whereas the color intensity represents the −log10 (FDR) of the enrichment analysis. BP: Biological processes; MF: Molecular function; CC: Cellular component. Analyses were limited to DEGs with Ensembl gene annotations. **(D)** Pathway analysis of DEGs from canine eyelid melanoma. The y-axis shows significantly enriched GO terms, and the x-axis indicates the fold enrichment. The size of the dots represents the number of genes associated with each term, and the colors of the dots represent the enrichment FDR (-log_10_). UP: upregulated DEGs; DOWN: downregulated DEGs.

After data standardization and batch effect removal, we identified 1,437 DEGs in the tumor samples. Of these, 672 genes were upregulated, and 765 genes were downregulated (FC ≥ 3, p < 0.05) (Table 1). The top five upregulated DEGs (logFC > 5.0) included *C3, STRA6, PPARG, MMP1*, and *NOS2*, whereas *MYL1, ACTA1, HBE1, LOC609402, and LOC476825* were the top five downregulated DEGs (logFC < −5.0) (Table 1). A detailed list of DEGs, including their corresponding fold changes and p-values, is provided in Supplementary Table S1. Hierarchical clustering of these DEGs showed a clear separation between normal and tumor tissues (Figure 1A).

**Table 1:** Characteristics of the 30 most differentially expressed genes in eyelid melanoma and their human orthologs, sorted by fold change (log FC ≥ 3.5 or logFC < −3.5 and p < 0.05).

	**Gene (Official Symbol)**	**Chromosome (Canine)**	**Chromosome (Human orthologs)**	**Description**	**logFC**
**Upregulated DEGs**	*C3*	chr20	chr19	Complement C3	6.92
	*STRA6*	chr30	chr15	Signaling receptor and transporter of retinol STRA6	6.87
	*PPARG*	chr20	chr3	Peroxisome proliferator activated receptor gamma	6.72
	*MMP1*	chr5	chr11	Matrix metallopeptidase 1	5.99
	*NOS2*	chr9	chr17	Nitric oxide synthase 2	5.49
	*CHI3L1*	chr7	chr1	Chitinase 3 like 1	5.38
	*APOC1*	chr1	chr19	Apolipoprotein C1	5.11
	*MMP9*	chr24	chr20	Matrix metallopeptidase 9	4.83
	*TTC29*	chr15	chr4	Tetratricopeptide repeat domain 29	4.78
	*LYZF2*	chr27	**Absent**	Lysozyme C, milk isozyme-like	4.71
	*VIL1*	chr37	chr2	Villin 1	4.69
	*PTGS2*	chr7	chr1	Prostaglandin-endoperoxide synthase 2	4.68
	*TMEM144*	chr15	chr4	Transmembrane protein 144	4.65
	*IGFBP2*	chr37	chr2	Insulin like growth factor binding protein 2	4.37
	*ACSL5*	chr28	chr10	Acyl-CoA synthetase long chain family member 5	4.31
	*KRT13*	chr9	chr17	Keratin 13	4.29
	*CIDEA*	chr7	chr18	Cell death inducing DFFA like effector a	4.29
	*SPEG*	chr37	chr2	Striated muscle enriched protein kinase	4.23
	*FBXO2*	chr2	ch1	F-box protein 2	4.17
	*VIT*	chr17	chr2	Vitrin	4.14
	*CFB*	chr12	chr6	Complement factor B	4.03
	*CRYBB1*	chr26	chr22	Crystallin beta B1	4.01
	*EDNRA*	chr15	chr4	Endothelin receptor type A	3.99
	*PLP1*	chrX	chrX	Proteolipid protein 1	3.83
	*FABP12*	chr29	chr8	Fatty acid binding protein 12	3.82
	*RASSF6*	chr13	chr4	Ras association domain family member 6	3.77
	*C1QC*	chr2	ch1	Complement C1q C chain	3.75
	*SIX1*	ch8	chr14	SIX homeobox 1	3.73
	*GATM*	chr30	chr15	Glycine amidinotransferase	3.63
	*PLAT*	chr16	chr8	Plasminogen activator, tissue type	3.60
	*LOC609288*	chr18	**Absent**	T cell receptor gamma variable 4	3.53
**Downregulated DEGs**	*MYL1*	chr37	chr2	Myosin light chain 1	-8.91
	*ACTA1*	chr4	chr1	Actin alpha 1, skeletal muscle	-8.26
	*HBE1*	chr21	chr11	Hemoglobin subunit epsilon 1	-7.96
	*LOC609402*	chr21	**Absent**	Hemoglobin subunit beta-like	-7.94
	*LOC476825*	chr21	**Absent**	Hemoglobin subunit beta	-7.63
	*KRT33A*	chr9	chr17	Keratin 33A	-7.26
	*MYL11*	chr6	chr16	myosin light chain 11	-7.20
	*MB*	chr10	chr22	Myoglobin	-7.13
	*KRT33B*	chr9	chr17	Keratin 33B	-7.12
	*TNNT3*	chr18	chr11	Troponin T3, fast skeletal type	-6.50
	*KRTAP11-1*	chr31	chr21	Keratin associated protein 11-1	-6.36
	*KRT34*	chr9	chr17	Keratin 34	-6.34
	*CKM*	chr1	chr19	Creatine kinase, M-type	-6.32
	*KRT26*	chr9	chr17	Keratin 26	-6.14
	*PGAM2*	chr16	chr7	Phosphoglycerate mutase 2	-5.46
	*PHC2*	chr2	chr1	Polyhomeotic homolog 2	-4.84
	*PRR9*	chr17	chr1	Proline rich 9	-4.79
	*MT4*	chr21	chr16	Metallothionein 4	-4.67
	*ATP2A1*	chr6	chr16	ATPase sarcoplasmic/endoplasmic reticulum Ca2+ transporting 1	-4.54
	*TTN*	chr36	chr2	Titin	-4.15
	*SLC5A9*	chr15	chr1	Solute carrier family 5 member 9	-4.12
	*CACNA1S*	chr7	chr1	Calcium voltage-gated channel subunit alpha1 S	-4.04
	*HOXA3*	chr14	chr3	Homeobox A3	-3.99
	*MYBPC1*	chr15	chr12	Myosin binding protein C1	-3.91
	*H19*	chr18	chr11	H19, imprinted maternally expressed transcript (non-protein coding)	-3.89
	*SLC39A4*	chr13	chr8	Solute carrier family 39 member 4	-3.88
	*TCAP*	chr9	chr17	Titin-cap	-3.83
	*MYL11*	chr11	chr16	Myosin light chain 11	-3.79
	*LOC100683419*	chr17	**Absent**	Myomegalin	-3.79
	*ACTN2*	chr4	chr1	Actinin alpha 2	-3.70
	*KRT31*	chr9	chr17	Keratin 31	-3.70

**In bold:** missing dog genes in humans.

To the best of our knowledge, this transcriptomic dataset represents the first systematic analysis focused on canine eyelid melanoma and provides evidence of the molecular drivers of its aggressive phenotype. Several top upregulated genes, including *MMP1*, *NOS2*, and *PPARG*, play well-established roles in extracellular matrix remodeling, immune modulation, and invasive growth. Notably, *MMP1* overexpression activates signaling pathways central to melanoma progression in humans, such as *Ras/Raf/MEK/ERK*, thereby enhancing cell proliferation and extracellular matrix degradation (Huntington et al., 2004; Iida & McCarthy, 2007). Similarly, *NOS2* has been implicated in metastatic dissemination through metabolic adaptation, enhanced survival, and immune cells recruitment (Douguet et al., 2016). Thus, both genes represent potential therapeutic targets in cases refractory to standard interventions. Simultaneously, the downregulation of cytoskeletal and adhesion-related genes such as *MYL1*, *ACTA1*, and *HBE1* may amplify invasiveness by compromising structural integrity and intercellular cohesion (Chen et al., 2021; Li et al., 2023).

### Enrichment analysis

Gene Ontology (GO) analysis of the DEGs revealed distinct transcriptional regulation patterns in canine eyelid melanoma. The upregulated genes were significantly enriched in immune-related processes, particularly cytokine signaling and G protein–coupled receptor activity (Figure 1B). This is consistent with previous evidence of immunosuppressive cytokines in canine melanomas, including IL-10 and TGF-β1 (Catchpole et al., 2002), which inhibit Th1 immune responses and promote regulatory T-cell differentiation (Takeuchi et al., 2021). Although tissue-level cytokine data in canine melanoma remain limited, cytokine-based immunotherapies such as intratumoral IL-2 and IL-12 combined with radiotherapy or gene transfection approaches have shown promising results in prolonging survival and eliciting antitumor responses (Dow et al., 1998; Stinson et al., 2024).

Conversely, the downregulated genes were associated with epidermal development, lipid metabolism, and extracellular matrix (ECM)-related functions, with significant depletion of glycosaminoglycan (GAG) binding and enzyme-inhibitory activities (Figure 1C). These changes indicate compromised ECM integrity, which may promote tumor progression and invasiveness (Brachelente et al., 2017; Smetsers et al., 2003). Consistent with this, metastatic melanoma cells often show reduced levels of heparan sulfate glycosaminoglycans (HS-GAGs) and proteoglycans such as versican (Kure et al., 1987; Seiz et al., 1990). Together, these findings highlight how immune evasion and ECM remodeling, partly mediated by *TGF-β1* signaling and the melanoma cell differentiation state, work together to define the malignant phenotype of canine eyelid melanoma (Serra et al., 2002).

### Pathway analysis

**Figure 2:**
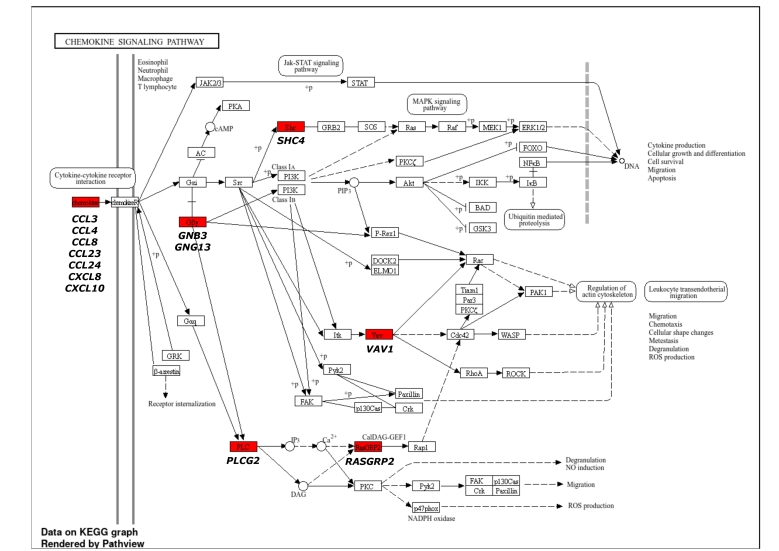
Upregulated DEGs in the chemokine signaling pathway in canine eyelid melanoma (KEGG analysis). Upregulated genes are highlighted in red boxes (*GNB3*, *CXCL8*, *CCL24*, *CCL4*, *CCL3*, *CCL8*, *CXCL10*, *CCL23*, *VAV1*, *SHC4*, *PLCG2*, *GNG13*, *RASGRP2*).

To define the biological pathways underlying canine eyelid melanoma, we performed KEGG-based functional annotation of DEGs (Kanehisa et al., 2012). This analysis revealed a strong enrichment of immune-related pathways among the upregulated DEGs, most prominently the chemokine signaling pathway (enrichment FDR = 1.517E^-06^) (Figures 1D and 2). Cancer cells express multiple chemokine receptors that respond directly to chemokines in the tumor microenvironment (Payne & Cornelius, 2002). Consistent with previous studies on human melanoma (Barrio-Alonso et al., 2024; Payne & Cornelius, 2002), canine eyelid melanoma showed high expression levels of *CCL3*, *CCL4*, *CCL8*, *CCL23*, *CCL24, CXCL8*, and *CXCL10* (Figure 2). These chemokines, typically observed in tumors with abundant T cell infiltration (often referred to as “hot tumors”) (Galon & Bruni, 2019), direct the trafficking of activated CD8^+^ T cells toward metastatic niches (Harlin et al., 2009). However, melanoma cells can also hijack this signaling pathway to recruit immunosuppressive populations that support their growth, survival, and metastasis while evading immune attacks (Adams et al., 2021).

Notably, our findings, along with those of recent studies, highlight the emerging roles of CCL8 and CCL23 in melanoma biology. CCL8, driven by macrophage–tumor interactions, amplifies an autocrine CCR1-driven loop that promotes the invasion, survival, and selection of metastasis-prone clones (Barbai et al., 2015; Barrio-Alonso et al., 2024). In parallel, CCL23—secreted by neutrophils, eosinophils, and monocytes—stimulates CCR1^+^ cancer cells, enhances the recruitment of immune and stromal cells into the tumor microenvironment (TME) (Meng et al., 2021), and induces immune evasion through the upregulation of checkpoint molecules, including CTLA-4, TIGIT, TIM-3, and LAG-3 (Kamat et al., 2022). Besides cancer, CCL23 has been implicated in inflammatory disorders (Castillo et al., 2010; Poposki et al., 2011) and promoting angiogenesis by enhancing VEGF signaling via KDR/Flk-1 in endothelial cells (Han et al., 2009). Collectively, CCL8 and CCL23 have emerged as key regulators of immune–tumor interactions and offer translational potential as therapeutic targets for canine eyelid melanoma.

Conversely, downregulated DEGs were enriched in “metabolic processes” and “estrogen signaling” (Figure 1D). The estrogen axis has recently been identified as therapeutically valuable in melanoma, given that the loss of estrogen receptor β (ERβ) and impairment of G-protein–coupled estrogen receptor (GPER) signaling correlate with immune evasion and poor outcomes (Giorgi et al., 2011; Natale et al., 2018). Although studies on canine eyelid melanoma are limited, studies on human cutaneous melanoma suggest that the disruption of estrogen axis signaling may contribute to disease progression and represent a potential avenue for therapeutic intervention (Marzagalli et al., 2016; Ribeiro et al., 2015).

### Canine eyelid melanoma is predicted to be associated with miRNA deregulation

**Figure 3:**
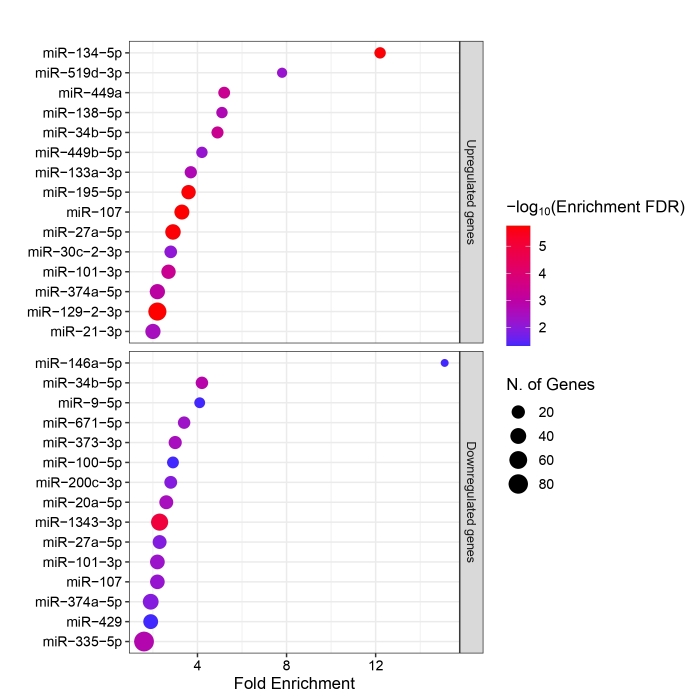
Scatterplots of the main miRNAs associated with upregulated and downregulated DEGs. The y-axis indicates significantly enriched miRNAs, and the x-axis indicates fold enrichment. The size of the dots represents the number of genes associated with each miRNA, and the colors of the dots represent the enrichment FDR (-log_10_). miRNAs are ordered by fold enrichment value.

Canine melanomas are strongly associated with miRNA dysregulation (Zamarian et al., 2019). Recent evidence has highlighted high sequence conservation between canine and mature human miRNAs (Leonardi et al., 2021). However, the lack of canine-specific miRNA databases (Varvil & Dos Santos, 2023) warrants the use of human prediction tools, such as DIANA-TarBase v8 (Karagkouni et al., 2018), to map interactions between DEGs and miRNAs in canine eyelid melanoma. Notably, *miR-134-5p* showed the highest enrichment among the miRNAs linked to the upregulated DEGs (fold enrichment = 12.15), whereas *miR-146a-5p* was the most enriched among those associated with the downregulated DEGs (fold enrichment = 15.1) (Figure 3). Further validation using CancerMIRNome and TCGA databases confirmed their diagnostic relevance in various human cancers (Supplementary Figure 1).

Mechanistic studies showed that *miR-134-5p* exhibits context-dependent activity. In melanoma, it is generally tumor-suppressive. Its inhibition by circRNA_0084043 promotes proliferation, invasion, and immune evasion, whereas its restoration reduces malignancy and improves therapeutic response (Cai et al., 2022). However, in lung cancer, it may enhance metastasis and chemoresistance (Zhang et al., 2019). Similarly, *miR-146a-5p* has dual roles, supporting melanoma cell growth via *NOTCH/PTEN/Akt* regulation and inhibiting metastasis depending on the tumor stage and microenvironment (Raimo et al., 2016). Additionally, genetic polymorphisms influence oncogenic or suppressive potential (Forloni et al., 2014). Together, these findings illustrate the complex, context-specific regulation mediated by *miR‑134‑5p* and *miR‑146a‑5p*, underscoring their translational value in canine and human melanoma.

**Figure 4:**
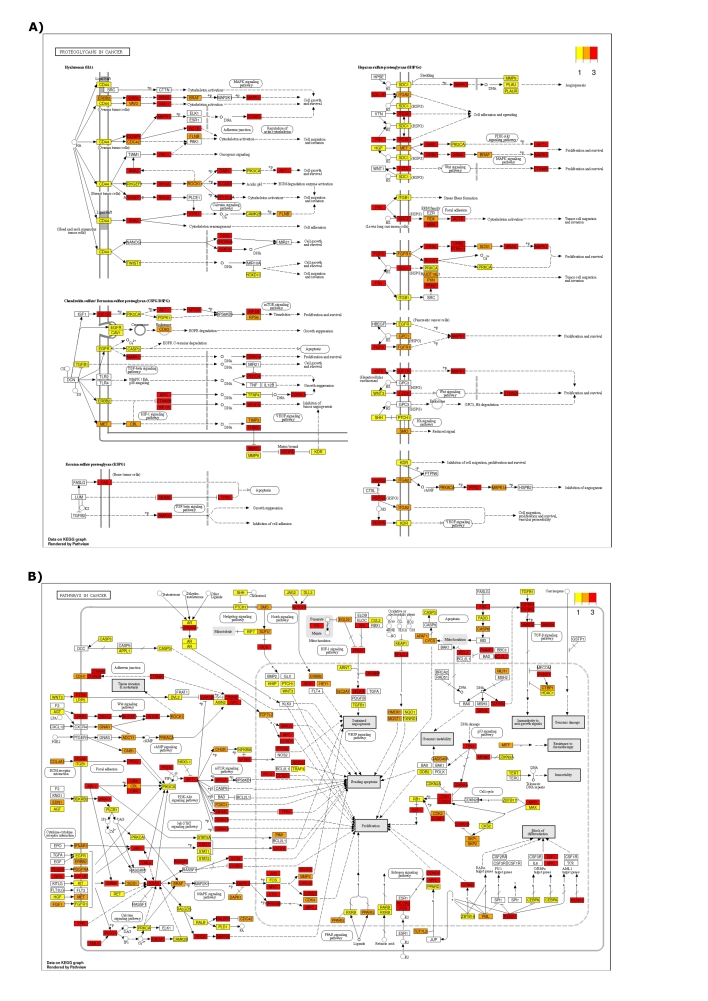
Enrichment of the most important oncogenic pathways in the KEGG analysis. The DIANA-miRPath v4.0 tool was used to identify KEGG pathways enriched with genes significantly targeted by the top predicted miRNAs. **(A)** Proteoglycans in cancer and **(B)** Pathways in cancer. Targets are highlighted according to the number of miRNAs targeting them (yellow: 1, orange: 2, red: ≥3).

To define the pathways potentially influenced by the deregulated miRNAs, we performed a functional analysis using the DIANA-miRPath (v4.0) tool (Tastsoglou et al., 2023). We found significant associations with oncogenic pathways, such as “proteoglycans in cancer” (Figure 4A) and “pathways in cancer” (Figure 4B) (Table 2). These findings indicated that deregulated miRNAs converge on conserved molecular networks that drive tumor progression.

**Table 2:** Enriched KEGG pathways for the main miRNAs associated with upregulated and downregulated DEGs in canine eyelid melanoma.

**KEGG Term**	**No. of miRNAs**	**No. of target genes**	**miRNAs**	***p*-value**	**FDR**
Ubiquitin mediated proteolysis	21	100	hsa-miR-100-5p, hsa-miR-101-3p, hsa-miR-107, hsa-miR-129-2-3p, hsa-miR-133a-3p, hsa-miR-1343-3p, hsa-miR-146a-5p, hsa-miR-195-5p, hsa-miR-200c-3p, hsa-miR-20a-5p, hsa-miR-21-3p, hsa-miR-27a-5p, hsa-miR-30c-2-3p, hsa-miR-335-5p, hsa-miR-34b-5p, hsa-miR-374a-5p, hsa-miR-429, hsa-miR-449a, hsa-miR-519d-3p, hsa-miR-671-5p, hsa-miR-9-5p	1.00e-18	3.43e-16
Cell cycle	22	88	hsa-miR-100-5p, hsa-miR-101-3p, hsa-miR-107, hsa-miR-129-2-3p, hsa-miR-133a-3p, hsa-miR-1343-3p, hsa-miR-146a-5p, hsa-miR-195-5p, hsa-miR-200c-3p, hsa-miR-20a-5p, hsa-miR-21-3p, hsa-miR-27a-5p, hsa-miR-30c-2-3p, hsa-miR-335-5p, hsa-miR-34b-5p, hsa-miR-373-3p, hsa-miR-374a-5p, hsa-miR-429, hsa-miR-449a, hsa-miR-519d-3p, hsa-miR-671-5p, hsa-miR-9-5p	2.76e-15	4.71e-13
Pathways in cancer	23	273	hsa-miR-100-5p, hsa-miR-101-3p, hsa-miR-107, hsa-miR-129-2-3p, hsa-miR-133a-3p, hsa-miR-134-5p, hsa-miR-1343-3p, hsa-miR-146a-5p, hsa-miR-195-5p, hsa-miR-200c-3p, hsa-miR-20a-5p, hsa-miR-21-3p, hsa-miR-27a-5p, hsa-miR-30c-2-3p, hsa-miR-335-5p, hsa-miR-34b-5p, hsa-miR-373-3p, hsa-miR-374a-5p, hsa-miR-429, hsa-miR-449a, hsa-miR-519d-3p, hsa-miR-671-5p, hsa-miR-9-5p	6.54e-14	7.43e-12
Proteoglycans in cancer	23	128	hsa-miR-100-5p, hsa-miR-101-3p, hsa-miR-107, hsa-miR-129-2-3p, hsa-miR-133a-3p, hsa-miR-134-5p, hsa-miR-1343-3p, hsa-miR-146a-5p, hsa-miR-195-5p, hsa-miR-200c-3p, hsa-miR-20a-5p, hsa-miR-21-3p, hsa-miR-27a-5p, hsa-miR-30c-2-3p, hsa-miR-335-5p, hsa-miR-34b-5p, hsa-miR-373-3p, hsa-miR-374a-5p, hsa-miR-429, hsa-miR-449a, hsa-miR-519d-3p, hsa-miR-671-5p, hsa-miR-9-5p	2.04e-13	1.74e-11
Autophagy - animal	22	93	hsa-miR-100-5p, hsa-miR-101-3p, hsa-miR-107, hsa-miR-129-2-3p, hsa-miR-133a-3p, hsa-miR-1343-3p, hsa-miR-146a-5p, hsa-miR-195-5p, hsa-miR-200c-3p, hsa-miR-20a-5p, hsa-miR-21-3p, hsa-miR-27a-5p, hsa-miR-30c-2-3p, hsa-miR-335-5p, hsa-miR-34b-5p, hsa-miR-373-3p, hsa-miR-374a-5p, hsa-miR-429, hsa-miR-449a, hsa-miR-519d-3p, hsa-miR-671-5p, hsa-miR-9-5p	2.75e-13	1.87e-11

### DEGs are enriched for specific hallmarks of cancer

**Figure 5:**
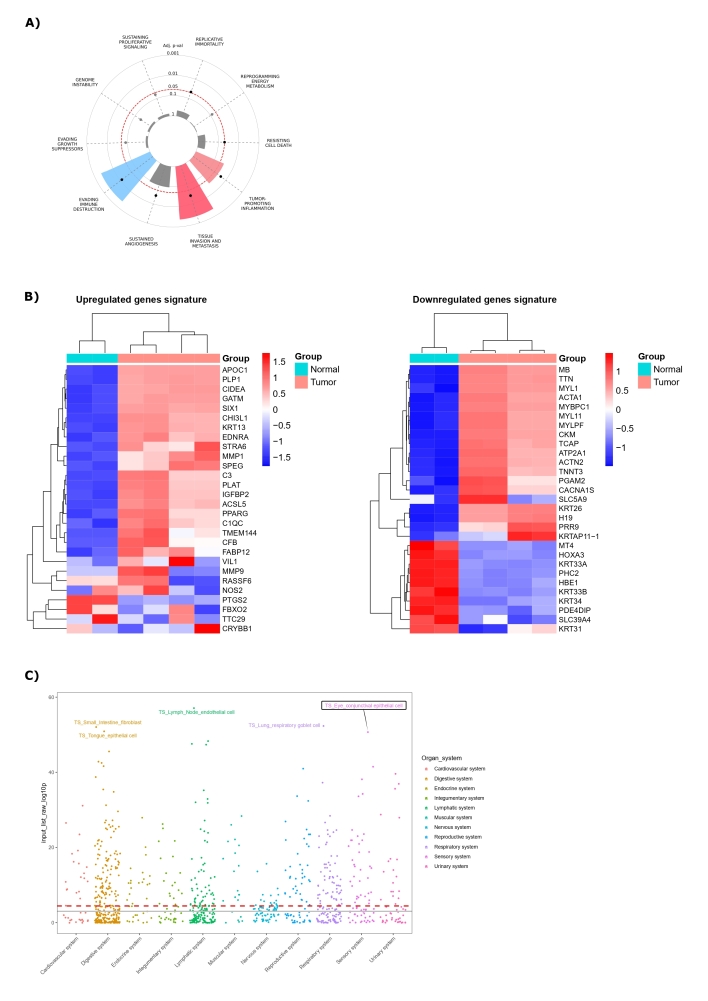
**(A)** Enrichment of cancer hallmarks in differentially expressed genes (DEGs) of canine eyelid melanoma. The graph shows the distribution of DEGs across ten established cancer hallmark categories, each depicted by a distinct color slice, with statistically significant enrichments (adjusted p < 0.05) highlighted. The slice size reflects the strength of enrichment compared to a reference gene set. Black and gray dots visually represent the distribution of genes: a black dot positioned closer to the outer border indicates a higher number of genes for a hallmark, while hallmarks without associated genes are indicated by gray dots near the inner border. **(B)** Cluster heatmap illustrating the expression profiles of the most DEGs from canine eyelid melanoma within the human eyelid basal cell carcinoma dataset (GSE103439). **Left:** Upregulated genes signature; **Right:** Downregulated genes signature. Both signatures showed conserved interspecies expression patterns. Hierarchical clustering was performed using the Euclidean distance metric and the complete linkage method, with gene-centric normalization performed prior to analysis. **(C)** Enrichment analysis using the WebCSEA tool showing the major organ system-specific cell signatures enriched in canine eyelid melanoma DEGs. Each dot represents an individual cell-type signature, color-coded by organ system. The y-axis indicates the significance of enrichment (log10 p-value) for each cell-type. The red dashed line marks the threshold for high statistical significance (p = 3.69 × 10^−5^). The solid gray line represents the nominal significance (p = 1 × 10^−3^).

Next, DEGs were analyzed to elucidate their role in the pathogenesis of canine eyelid melanoma. Using the CancerHallmarks database (https://cancerhallmarks.com/) (Menyhart et al., 2025), we found that DEGs were significantly enriched in hallmarks categories central to tumor progression. Specifically, enrichment was observed for immune evasion, tumor-promoting inflammation, tissue invasion, and metastasis (Figure 5A). These findings indicate that molecular alterations in canine eyelid melanoma converge on core oncogenic processes that drive disease aggressiveness.

### Cross-validation with human basal cell carcinoma of the eyelid

To evaluate cross-species transcriptional similarities, we compared the DEGs between canine eyelid melanoma and human eyelid basal cell carcinoma (BCC). We focused on the top 30 DEGs identified in canine melanoma and limited our analysis to genes with human homologs. Notably, most genes upregulated in canine eyelid melanoma (*APOC1*, *PLP1*, *CIDEA*, *GATM*, *SIX1*, *CHI3L1*, *KRT13*, *EDNRA*, *STRA6*, *MMP1*, *SPEG*, *C3*, *PLAT*, *IGFBP2*, *ACSL5*, *PPARG*, *C1QC*, *TMEM144*, *CFB*, and *FABP12*) were also upregulated in human eyelid BCC. In contrast, only a subset of the downregulated genes (*MT4*, *HOXA3*, *KRT33A*, *PHC2*, *HBE1*, *KRT33B*, *KRT34*, *PDE4DIP*, *SLC39A4*, and *KRT31*) showed cross-species concordance (Figure 5B). These differences likely reflect intrinsic variations in cellular origin, tissue composition, and the tumor microenvironment.

Comparative analyses are central to translational oncology, because dogs spontaneously develop mucosal melanomas that resemble the clinical, histological, and genetic features of human melanomas (Hernandez et al., 2018; Prouteau & Andre, 2019; Wong et al., 2019). Both species share recurrent alterations in driver genes such as *NRAS*, *TP53*, and *PTEN*, along with similar copy number alterations (Wong et al., 2019). Unlike human cutaneous melanomas, mucosal forms in both species rarely harbor *BRAF*, and *c-KIT* mutations, but instead show aberrant activation of the *AKT* and *MAPK* signaling pathways (Simpson et al., 2014). This cross-species approach provides a unique opportunity to investigate non-UV–driven melanomas and evaluate therapeutic strategies, including immunotherapies and inhibitors of the *RAS/MAPK* and *PI3K/AKT/mTOR* signaling pathways (Hernandez et al., 2018). However, incomplete canine genome annotation and limited sequencing depth remain the major barriers to fully leveraging this model (Hernandez et al., 2018).

### Cell type-specific enrichment analysis

Finally, we performed a cell type–specific enrichment analysis using the WebCSEA tool (Dai et al., 2022) to assess the tissue-specific expression patterns of the DEGs in canine eyelid melanoma. The analysis showed a significant enrichment of conjunctival epithelial cells (Figure 5C), consistent with the cellular origin of the tumor. This finding confirmed the cellular identity and anatomical origin of the samples, highlighting the relevance of this model in comparative oncology and underscoring its potential as a guide for discovering therapeutic targets.

## Conclusions

Our study revealed new aspects of the molecular landscape underlying malignant canine eyelid melanoma and identified the pathways and markers associated with its aggressive phenotype. Differential expression analysis showed that the upregulated genes were enriched in chemokine signaling, whereas the downregulated genes were linked to hormonal regulation. Additionally, *miR-134-5p* and *miR-146a-5p* have emerged as potential modulators of oncogenic pathways, including proteoglycan-mediated pathways, highlighting their diagnostic and therapeutic relevance. Although our exploratory pilot study was limited by its single-case design and relied on human BCC data owing to the lack of transcriptomic information for human eyelid melanoma, which differs in pathogenesis and biology from melanoma, our findings advance the understanding of canine melanoma biology and highlight promising directions for veterinary and comparative oncology.

## Supplementary Material

Supplementary material accompanies this paper.

Supplementary Figure 1 Receiver operating characteristic (ROC) analysis of miRNA expression to distinguish tumor and normal samples in The Cancer Genome Atlas (TCGA) data. (A) ROC for hsa-miR-134-5p and (B) miR-146a-5p across multiple cancer types. Area under the ROC curve (AUC) values with 95% confidence intervals are shown for each cancer type, indicating the diagnostic performance of each miRNA in distinguishing tumor from normal tissue. Sample sizes for tumor and normal cohorts are shown for each project. Red dots represent estimated AUC values; black horizontal lines indicate the corresponding confidence intervals. Higher AUC values reflect better discrimination between tumor and normal samples.

Supplementary Table S1 Total number of differentially expressed genes identified in the canine malignant eyelid melanoma in this study.

This material is available as part of the online article from https://doi.org/10.29374/2527-2179.bjvm004025
