# Supplementary material for: Transcriptomic profiling of a canine malignant eyelid melanoma
Source: Braz J Vet Med. 2025 Dec 12;48:e004025. doi: 10.29374/2527-2179.bjvm004025 (PMC12700502; doi:10.29374/2527-2179.bjvm004025)

A) ROC Analysis between tumor and normal samples from TCGA (hsa-miR-134-5p)

Project	Tumor	Normal	AUC (95% CI)
TCGA-KICH	66	25	0.91 (0.85–0.97)
TCGA-COAD	444	8	0.9 (0.84–0.96)
TCGA-CESC	307	3	0.9 (0.78–1)
TCGA-UCEC	538	33	0.89 (0.84–0.93)
TCGA-READ	161	3	0.88 (0.78–0.97)
TCGA-KIRP	291	34	0.85 (0.79–0.91)
TCGA-LIHC	372	50	0.78 (0.73–0.82)
TCGA-PRAD	494	52	0.77 (0.71–0.82)
TCGA-BLCA	409	19	0.77 (0.69–0.85)
TCGA-BRCA	1078	104	0.75 (0.71–0.79)
TCGA-THCA	506	59	0.7 (0.66–0.75)
TCGA-KIRC	516	71	0.7 (0.64–0.75)
TCGA-HNSC	523	44	0.69 (0.63–0.76)
TCGA-THYM	124	2	0.66 (0.46–0.85)
TCGA-LUAD	513	46	0.64 (0.58–0.7)
TCGA-ESCA	184	13	0.61 (0.48–0.75)
TCGA-PCPG	179	3	0.6 (0.39–0.82)
TCGA-LUSC	478	45	0.58 (0.51–0.66)
TCGA-SKCM	97	2	0.56 (0–1)
TCGA-CHOL	36	9	0.53 (0.37–0.69)
TCGA-STAD	436	41	0.5 (0.44–0.57)
TCGA-PAAD	178	4	0.44 (0.17–0.7)

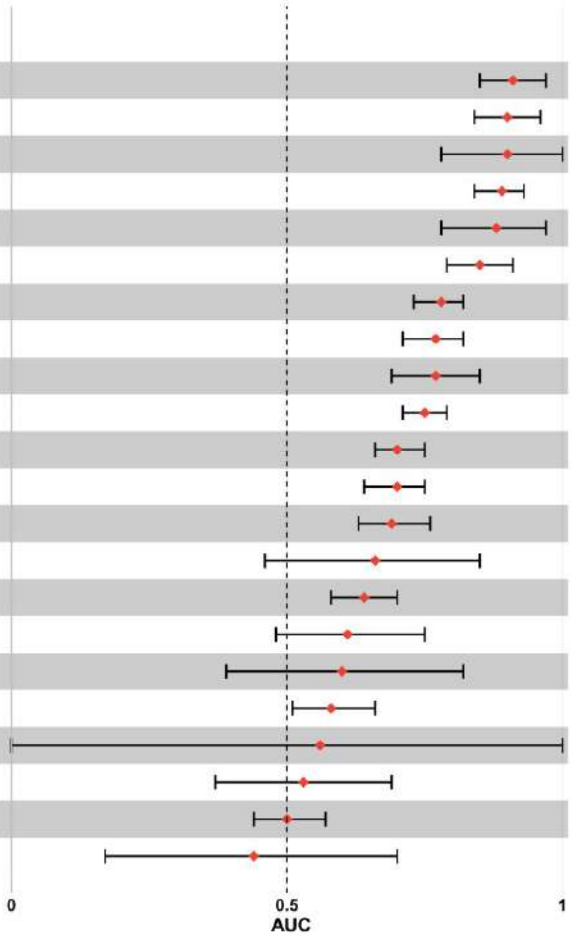

B) ROC Analysis between tumor and normal samples from TCGA (miR-146a-5p)

Project	Tumor	Normal	AUC (95% CI)
TCGA-READ	161	3	1 (1–1)
TCGA-SKCM	97	2	0.94 (0.83–1)
TCGA-COAD	444	8	0.93 (0.86–1)
TCGA-KIRP	291	34	0.87 (0.81–0.93)
TCGA-THYM	124	2	0.85 (0.75–0.95)
TCGA-KIRC	516	71	0.81 (0.76–0.85)
TCGA-ESCA	184	13	0.76 (0.61–0.91)
TCGA-UCEC	538	33	0.75 (0.67–0.82)
TCGA-CESC	307	3	0.75 (0.34–1)
TCGA-LIHC	372	50	0.65 (0.6–0.71)
TCGA-KICH	66	25	0.65 (0.52–0.78)
TCGA-PAAD	178	4	0.62 (0.32–0.93)
TCGA-STAD	436	41	0.58 (0.46–0.7)
TCGA-PCPG	179	3	0.57 (0.36–0.78)
TCGA-BRCA	1078	104	0.57 (0.52–0.62)
TCGA-PRAD	494	52	0.56 (0.47–0.65)
TCGA-LUSC	478	45	0.56 (0.49–0.63)
TCGA-HNSC	523	44	0.56 (0.47–0.66)
TCGA-LUAD	513	46	0.52 (0.43–0.6)
TCGA-CHOL	36	9	0.52 (0.35–0.69)
TCGA-THCA	506	59	0.5 (0.43–0.57)
TCGA-BLCA	409	19	0.5 (0.36–0.64)

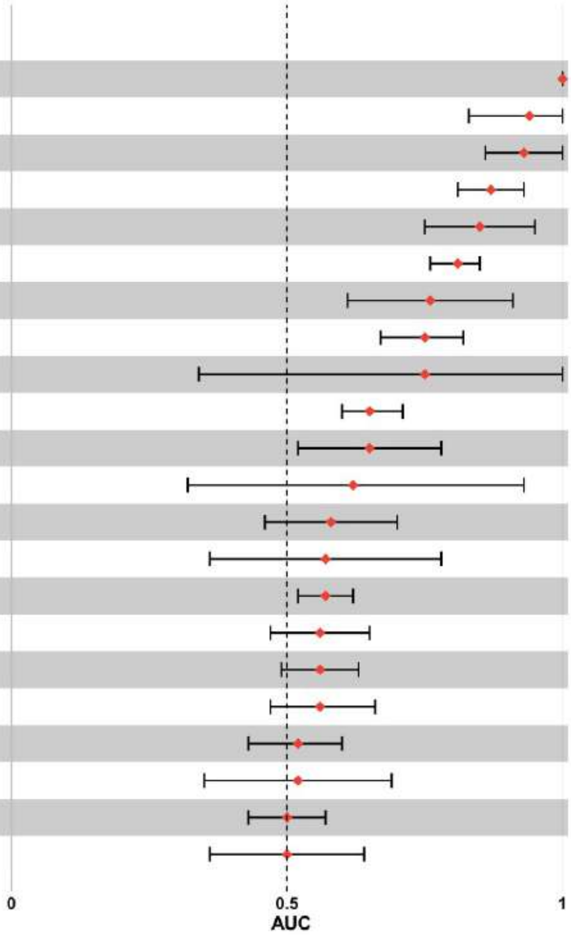

Supplement: Supplementary Figure 1 [file bjvm-48-e004025-suppl-g01.pdf]
