# Supplementary material for: Transcriptomic profiling of a canine malignant eyelid melanoma
Source: Braz J Vet Med. 2025 Dec 12;48:e004025. doi: 10.29374/2527-2179.bjvm004025 (PMC12700502; doi:10.29374/2527-2179.bjvm004025)
Supplement: Supplementary Table S1 [file bjvm-48-e004025-suppl-gt01.pdf]

ProbeName	FC ([Tumor] vs [Normal])	Log FC ([Tumor] vs [Normal])	FC (abs) ([Tumor] vs [Normal])	Regulation ([Tumor] vs [Normal])	GeneSymbol	Chromosome Number_Avadis_
A_11_P052641	3.447695	1.7856321	3.447695	up	DLA-DQB1	chr12
A_11_P0000015451	9.570811	3.2586412	9.570811	up	GATM	chr30
A_11_P0000030446	3.7579975	1.9099641	3.7579975	up		chr27
A_11_P0000028827	-5.5751257	-2.4790044	5.5751257	down	SHISA5	chr20
A_11_P0000014584	-4.5315633	-2.180009	4.5315633	down	DEFB1	chr16
A_11_P0000019501	6.5731883	2.7165933	6.5731883	up	GAL	chr18
A_11_P069376	-5.122228	-2.3567715	5.122228	down		chr21
A_11_P152618	6.983482	2.8039465	6.983482	up	IGF2	chr18
A_11_P148368	-301.61325	-8.236556	301.61325	down	MYL1	chr37
A_11_P060201	-3.56325	-1.8331938	3.56325	down		chr26
A_11_P055696	3.0133958	1.5913901	3.0133958	up	PBX1	chr38
A_11_P0000020106	3.8580215	1.9478612	3.8580215	up	CCL8	chr9
A_11_P0000016202	3.5610027	1.8322835	3.5610027	up		chr27
A_11_P107736	-3.659825	-1.8717747	3.659825	down	GPR157	chr5
A_11_P050666	25.716404	4.684617	25.716404	up	PTGS2	chr7
A_11_P0000023925	-3.9012728	-1.9639449	3.9012728	down	ITPRID2	chr36
A_11_P0000033278	4.43172	2.1478667	4.43172	up	LRRC8D	chr6
A_11_P152143	3.605228	1.8500905	3.605228	up	MLXIPL	chr6
A_11_P077906	3.2211063	1.6875563	3.2211063	up	RARRES2	chr16
A_11_P0000032442	3.5985363	1.8474102	3.5985363	up	ESM1	chr4
A_11_P0000030339	-3.5924044	-1.8449497	3.5924044	down	AMIGO2	chr27
A_11_P069586	3.0945308	1.6297207	3.0945308	up	SERGEF	chr21
A_11_P0000023492	-3.5037951	-1.8089185	3.5037951	down	FBN1	chr30
A_11_P060506	4.9993286	2.3217344	4.9993286	up	DEPDC5	chr26
A_11_P0000025269	-8.140691	-3.0251513	8.140691	down		chr8
A_11_P0000019760	3.2131016	1.6839666	3.2131016	up	SOD1	chr31

A_11_P062231	4.1660237	2.058671	4.1660237	up	SECISBP2L	chr30
A_11_P0000023370	4.7846513	2.2584138	4.7846513	up	FABP3	chr2
A_11_P166633	106.04959	6.7285953	106.04959	up		chr20
A_11_P201423	3.1962497	1.6763802	3.1962497	up	DAB2	chr4
A_11_P109066	-4.398503	-2.1370125	4.398503	down	CERS3	chr3
A_11_P0000038891	12.859754	3.684791	12.859754	up		chr33
A_11_P169713	4.4888296	2.1663394	4.4888296	up		chr8
A_11_P0000034661	3.237383	1.694828	3.237383	up	ERAS	chrX
A_11_P0000020089	-3.769082	-1.9142132	3.769082	down	KRT2	chr27
A_11_P082456	4.6313457	2.2114315	4.6313457	up	CCL14	chr9
A_11_P0000019268	-3.1294565	-1.6459122	3.1294565	down		chr9
A_11_P083011	3.3623586	1.7494736	3.3623586	up	NSMF	chr9
A_11_P0000025489	-3.959319	-1.9852524	3.959319	down	CLIC3	chr9
A_11_P058311	-4.3980813	-2.1368742	4.3980813	down	TMEM45A	chr33
A_11_P140731	-3.3706636	-1.7530327	3.3706636	down	LOC607776	chrX
A_11_P0000031482	-6.4245768	-2.6836014	6.4245768	down	CSTA	chr33
A_11_P138501	6.00358	2.585823	6.00358	up		
A_11_P197573	6.274147	2.6494193	6.274147	up		chr9
A_11_P183038	9.578944	3.2598667	9.578944	up	GATM	chr30
A_11_P164513	4.017981	2.0064707	4.017981	up	TEX2	chr9
A_11_P0000019770	-3.5674028	-1.8348742	3.5674028	down	TMEM47	chrX
A_11_P197298	-3.434881	-1.7802601	3.434881	down		chr16
A_11_P0000018997	3.1321657	1.6471605	3.1321657	up		chr5
A_11_P0000029457	-3.304939	-1.7246237	3.304939	down	ABHD5	chr23
A_11_P0000013221	-3.000696	-1.5852971	3.000696	down		chr17
A_11_P0000014629	9.866458	3.3025322	9.866458	up		chr6
A_11_P078161	3.0445597	1.6062336	3.0445597	up	PLAT	chr16
A_11_P0000039420	-3.9072359	-1.9661484	3.9072359	down		chr22

A_11_P0000022922	-4.445522	-2.1523528	4.445522	down		chr27
A_11_P051016	5.3365993	2.4159207	5.3365993	up	IL13RA2	chrX
A_11_P149338	-17.52139	-4.1310453	17.52139	down	PGAM2	chrUn_JH373336
A_11_P0000031571	-3.8092659	-1.929513	3.8092659	down	LOC102152005	chr34
A_11_P0000024694	-3.793746	-1.9236231	3.793746	down	AZGP1	chr6
A_11_P0000017392	5.9607024	2.5754824	5.9607024	up		chr2
A_11_P192543	3.5271325	1.8184958	3.5271325	up	APOE	chr1
A_11_P0000016593	10.48474	3.3902192	10.48474	up	TTYH1	chr1
A_11_P053416	3.4172409	1.7728319	3.4172409	up	CXCL8	chr13
A_11_P0000031595	-3.6216629	-1.8566523	3.6216629	down	PPM1L	chr34
A_11_P0000024453	-4.2216988	-2.0778236	4.2216988	down	ENO3	chr5
A_11_P000009106	-5.978972	-2.5798974	5.978972	down		chr13
A_11_P0000024710	-4.193389	-2.0681167	4.193389	down	LOC479761	chr6
A_11_P051251	-5.625101	-2.491879	5.625101	down	KRT12	chr9
A_11_P110201	-3.046587	-1.607194	3.046587	down	SERPINB5	chr1
A_11_P000006383	-3.5277038	-1.8187294	3.5277038	down		
A_11_P057906	-4.8327546	-2.2728457	4.8327546	down	ABCA12	chr37
A_11_P0000034188	4.602566	2.2024384	4.602566	up	DIPK1B	chr9
A_11_P106336	63.90129	5.997773	63.90129	up	MMP1	chr5
A_11_P0000029880	4.289849	2.1009269	4.289849	up	HPGD	chr25
A_11_P0000031934	4.9079294	2.2951145	4.9079294	up	TGFB2	chr38
A_11_P0000039279	-4.5239835	-2.1775937	4.5239835	down	LOC478001	chr2
A_11_P090541	-4.9005527	-2.2929444	4.9005527	down	ENPP3	chr12
A_11_P107456	10.505858	3.3931222	10.505858	up	TNFRSF18	chr5
A_11_P000004046	5.6061544	2.4870114	5.6061544	up		chr2
A_11_P0000026463	-3.8382983	-1.9404669	3.8382983	down	ELOVL4	chr12
A_11_P108001	9.248121	3.2091603	9.248121	up	PLCG2	chr5
A_11_P0000030745	-9.682277	-3.2753463	9.682277	down	BAMBI	chr2

A_11_P114506	3.0573077	1.6122618	3.0573077	up	CASK	chrX
A_11_P149413	-4.656392	-2.2192125	4.656392	down		chr25
A_11_P098171	-5.1257067	-2.357751	5.1257067	down		chr6
A_11_P0000030608	-5.814404	-2.5396314	5.814404	down	EMX2	chr28
A_11_P000003754	3.1972694	1.6768403	3.1972694	up		chr24
A_11_P127286	3.173613	1.6661263	3.173613	up	TP73	chr5
A_11_P0000032142	-3.7934225	-1.9235001	3.7934225	down	KLF3	chr3
A_11_P098371	-5.239756	-2.3894997	5.239756	down	CTH	chr6
A_11_P056091	6.5540605	2.712389	6.5540605	up	GMDS	chr35
A_11_P0000014339	3.3284762	1.7348619	3.3284762	up	SCD	chr28
A_11_P213013	-5.1285214	-2.358543	5.1285214	down		chr12
A_11_P064686	3.1618876	1.6607862	3.1618876	up	SEMA5A	chr34
A_11_P125021	3.013109	1.5912528	3.013109	up		chr4
A_11_P0000039078	-5.4061832	-2.4346104	5.4061832	down		chr25
A_11_P050096	-3.0815747	-1.6236677	3.0815747	down	TMEM47	chrX
A_11_P059826	-5.0386205	-2.3330288	5.0386205	down	HCAR2	chr26
A_11_P0000020059	5.228935	2.386517	5.228935	up	LAMC2	chr7
A_11_P0000029648	3.2453194	1.6983604	3.2453194	up	CPXM1	chr24
A_11_P059271	3.7687619	1.9140906	3.7687619	up	SNCA	chr32
A_11_P0000034272	3.5589893	1.8314676	3.5589893	up	NEK6	chr9
A_11_P0000014989	4.715133	2.2372985	4.715133	up	CCL24	chr6
A_11_P121921	4.1266837	2.044983	4.1266837	up		chr18
A_11_P073221	7.407935	2.8890715	7.407935	up	RCN1	chr18
A_11_P081631	-139.72263	-7.126422	139.72263	down	KRT33B	chr9
A_11_P0000021557	5.839945	2.5459547	5.839945	up	RCN1	chr18
A_11_P0000017935	-3.0872223	-1.6263094	3.0872223	down		chr28
A_11_P069726	-7.00087	-2.8075342	7.00087	down	MUC15	chr21
A_11_P190608	5.214779	2.382606	5.214779	up		chr4

A_11_P0000033174	-3.3669393	-1.7514377	3.3669393	down	ZSCAN10	chr6
A_11_P0000029369	-10.999237	-3.4593315	10.999237	down	KCTD4	chr22
A_11_P0000026507	4.960091	2.3103666	4.960091	up	BEND3	chr12
A_11_P051036	3.3035738	1.7240276	3.3035738	up	EMR4	chr20
A_11_P056961	-3.2754688	-1.7117014	3.2754688	down	DPP4	chr36
A_11_P072341	-3.0410528	-1.6045709	3.0410528	down	TFCP2L1	chr19
A_11_P000002831	3.116518	1.639935	3.116518	up	ABCA1	chr11
A_11_P065996	-8.604939	-3.105165	8.604939	down	IRAG2	chr27
A_11_P0000017998	3.1197674	1.6414385	3.1197674	up		chr24
A_11_P0000021828	-3.0633392	-1.6151052	3.0633392	down	RSPO3	chr1
A_11_P105476	3.2763696	1.7120981	3.2763696	up		chr5
A_11_P217703	-44.04847	-5.46102	44.04847	down	PGAM2	chrUn_JH373336
A_11_P00000865	-6.087069	-2.6057477	6.087069	down	CARM1	chr20
A_11_P0000027549	3.0677924	1.6172009	3.0677924	up	EGFR	chr18
A_11_P166853	8.063957	3.011488	8.063957	up		chr17
A_11_P097641	4.8182616	2.2685127	4.8182616	up	MSRB1	chr6
A_11_P0000024439	-3.8440852	-1.9426403	3.8440852	down	GUCY1A2	chr5
A_11_P085986	5.8936143	2.5591526	5.8936143	up	DUSP6	chr15
A_11_P079306	5.5652375	2.4764433	5.5652375	up	ETV1	chr14
A_11_P0000026808	-3.1782928	-1.668252	3.1782928	down	SOSTDC1	chr14
A_11_P0000021647	-5.925424	-2.5669184	5.925424	down	SCGB1A1	chr18
A_11_P148363	4.1448092	2.0513058	4.1448092	up		chr9
A_11_P0000033733	-4.4202805	-2.1441379	4.4202805	down	DAAM1	chr8
A_11_P069791	5.202287	2.379146	5.202287	up	MS4A7	chr21
A_11_P0000013643	-3.2399166	-1.6959567	3.2399166	down		chr20
A_11_P179123	3.8895423	1.9596004	3.8895423	up		chr3
A_11_P126726	5.2022686	2.3791409	5.2022686	up		chr1
A_11_P107926	-3.247987	-1.6995459	3.247987	down	KIAA0513	chr5

A_11_P0000020093	-4.318693	-2.1105947	4.318693	down	KRT1	chr27
A_11_P0000031639	3.2921367	1.7190242	3.2921367	up	GMDS	chr35
A_11_P0000023795	-3.1980727	-1.6772027	3.1980727	down	FETUB	chr34
A_11_P097921	-3.061747	-1.6143551	3.061747	down	ELAPOR1	chr6
A_11_P0000027165	4.896421	2.2917275	4.896421	up	RAB19	chr16
A_11_P0000013647	-3.0931044	-1.6290555	3.0931044	down		
A_11_P071611	-8.201933	-3.035964	8.201933	down	ACKR4	chr23
A_11_P158328	3.2255447	1.6895428	3.2255447	up	ARHGEF28	chr2
A_11_P107871	3.6399565	1.8639212	3.6399565	up	SLC7A5	chr5
A_11_P0000041102	3.6093915	1.8517556	3.6093915	up		chrUn_JH373316
A_11_P0000034346	-3.927569	-1.9736366	3.927569	down	LY6D	chrUn_JH373372
A_11_P134501	-4.2022977	-2.0711784	4.2022977	down		chr12
A_11_P059406	-5.0833797	-2.345788	5.0833797	down	SLC39A8	chr32
A_11_P091771	-3.6919847	-1.8843966	3.6919847	down		chr12
A_11_P0000034085	-3.717208	-1.8942194	3.717208	down	DUSP14	chr9
A_11_P0000019114	3.0600595	1.6135597	3.0600595	up		chr5
A_11_P0000038911	-4.209674	-2.0737085	4.209674	down		chr30
A_11_P105456	32.304417	5.0136595	32.304417	up		chr5
A_11_P122951	7.1339664	2.8347044	7.1339664	up	DLC1	chr16
A_11_P051646	26.246683	4.714063	26.246683	up	LYZF2	chr27
A_11_P190653	3.3627653	1.7496481	3.3627653	up		chr1
A_11_P0000030776	-4.472325	-2.161025	4.472325	down	NMT2	chr2
A_11_P0000033212	9.577216	3.2596064	9.577216	up	GNG13	chr6
A_11_P000006343	20.175583	4.3345385	20.175583	up		chr18
A_11_P0000031538	-3.1787958	-1.6684804	3.1787958	down	TPPP	chr34
A_11_P186938	-3.3799365	-1.7569962	3.3799365	down	NIBAN1	chr7
A_11_P205298	8.991304	3.1685305	8.991304	up		chr15
A_11_P0000024739	-23.380247	-4.5472183	23.380247	down	ATP2A1	chr6

A_11_P173368	4.770208	2.2540522	4.770208	up		chr7
A_11_P099136	-4.1698613	-2.0599995	4.1698613	down	FMO2	chr7
A_11_P0000027350	4.736144	2.243713	4.736144	up	CGREF1	chr17
A_11_P076161	3.2712526	1.7098432	3.2712526	up	CRLF1	chr20
A_11_P0000015816	-9.291794	-3.2159572	9.291794	down		chr2
A_11_P162053	4.1450615	2.0513935	4.1450615	up		chr16
A_11_P170543	-5.5520535	-2.4730215	5.5520535	down		chr9
A_11_P0000034446	6.1575804	2.6223636	6.1575804	up	PDGFA	chr6
A_11_P0000016626	-4.8765616	-2.2858644	4.8765616	down		chr28
A_11_P174428	-5.3797474	-2.4275384	5.3797474	down		chr32
A_11_P0000023949	-7.177375	-2.8434563	7.177375	down	TMEFF2	chr37
A_11_P0000016911	-6.4832954	-2.6967273	6.4832954	down		chr26
A_11_P172128	3.4948826	1.805244	3.4948826	up		chr16
A_11_P052916	-3.4736228	-1.7964411	3.4736228	down	DSG1	chr7
A_11_P064896	7.2562914	2.8592324	7.2562914	up	CLCN2	chr34
A_11_P0000033068	-3.4509509	-1.786994	3.4509509	down	PYCARD	chr6
A_11_P0000016891	-3.0281563	-1.5984397	3.0281563	down		chrX
A_11_P174433	-3.524677	-1.817491	3.524677	down		chr22
A_11_P0000031096	-7.093715	-2.8265414	7.093715	down		chr30
A_11_P179173	3.35676	1.7470694	3.35676	up		chr9
A_11_P085481	-7.181962	-2.844378	7.181962	down	LOC475367	chr15
A_11_P0000041529	3.1194263	1.6412807	3.1194263	up	ART3	chr32
A_11_P106346	-5.7080636	-2.5130014	5.7080636	down	MMP7	chr5
A_11_P138641	-64.89205	-6.01997	64.89205	down		chr24
A_11_P0000015158	-3.2101922	-1.6826596	3.2101922	down		
A_11_P0000019993	5.7951646	2.5348496	5.7951646	up	ADORA1	chr7
A_11_P0000034181	5.202958	2.379332	5.202958	up		chr9
A_11_P0000040120	-5.889521	-2.5581503	5.889521	down	MARCHF3	chr11

A_11_P115356	3.9602091	1.9855766	3.9602091	up		chrX
A_11_P000001229	-3.8294005	-1.9371185	3.8294005	down		
A_11_P00000115	-4.1183257	-2.042058	4.1183257	down	VLDLR	chr1
A_11_P0000015621	-3.209252	-1.6822371	3.209252	down		chr2
A_11_P000009018	-3.6892474	-1.8833265	3.6892474	down		chr15
A_11_P059411	3.6337047	1.8614411	3.6337047	up	SLC9B1	chr32
A_11_P0000030927	-3.604138	-1.8496542	3.604138	down		chr2
A_11_P0000022671	-6.0181108	-2.5893106	6.0181108	down	POSTN	chr25
A_11_P0000040801	3.33496	1.7376695	3.33496	up		chr17
A_11_P0000020166	4.8205333	2.2691927	4.8205333	up	CXCL10	chr32
A_11_P0000040669	-3.160729	-1.6602573	3.160729	down	MYOM1	chr7
A_11_P0000029946	-4.4468164	-2.152773	4.4468164	down	LOC486151	chr25
A_11_P128391	-3.9591386	-1.9851866	3.9591386	down		chr33
A_11_P065446	-8.652475	-3.113113	8.652475	down	KRT73	chr27
A_11_P0000025459	-4.119671	-2.042529	4.119671	down	BLMH	chr9
A_11_P0000020551	-4.0479527	-2.0171924	4.0479527	down	DNAI1	chr11
A_11_P0000029161	-4.8514924	-2.2784286	4.8514924	down	DLG2	chr21
A_11_P179698	-3.9490664	-1.9815116	3.9490664	down	GALNT14	chr17
A_11_P0000030169	3.966817	1.9879818	3.966817	up	GGT1	chr26
A_11_P0000030711	-4.080989	-2.0289187	4.080989	down	MMP16	chr29
A_11_P160268	-259.7931	-8.021219	259.7931	down	ACTA1	chr4
A_11_P128171	3.842411	1.9420118	3.842411	up	ART3	chr32
A_11_P107911	-3.1846535	-1.6711364	3.1846535	down	C5H16orf74	chr5
A_11_P085781	-3.1222162	-1.6425705	3.1222162	down	RNASE4	chr15
A_11_P071681	-3.4593046	-1.790482	3.4593046	down	IL20RB	chr23
A_11_P0000028448	-4.021393	-2.0076952	4.021393	down	KLK9	chr1
A_11_P089691	-3.6569376	-1.870636	3.6569376	down	TCP11L2	chr10
A_11_P117651	-8.820874	-3.1409216	8.820874	down		chr14

A_11_P0000015569	-5.351088	-2.4198322	5.351088	down		
A_11_P188048	4.1343617	2.0476646	4.1343617	up		chr1
A_11_P000002722	-4.417089	-2.143096	4.417089	down		
A_11_P0000031385	4.177893	2.0627756	4.177893	up	DDIT4L	chr32
A_11_P058316	-4.1919312	-2.067615	4.1919312	down	ABI3BP	chr33
A_11_P114961	-3.191934	-1.6744308	3.191934	down	GDPD2	chrX
A_11_P0000020054	3.6243405	1.8577185	3.6243405	up	LGALS9	chr9
A_11_P137521	4.8524046	2.2786999	4.8524046	up		chr12
A_11_P0000029111	-9.088775	-3.1840858	9.088775	down		chr20
A_11_P090621	-6.8722486	-2.7807822	6.8722486	down	CDSN	chr12
A_11_P0000027376	14.830809	3.8905253	14.830809	up	VIT	chr17
A_11_P0000022713	-4.697281	-2.2318258	4.697281	down	ATP12A	chr25
A_11_P192353	3.4510262	1.7870255	3.4510262	up		chr18
A_11_P111176	3.0687275	1.6176405	3.0687275	up	TMEM200A	chr1
A_11_P0000041309	-10.777233	-3.429915	10.777233	down		chr22
A_11_P0000016374	10.565911	3.4013453	10.565911	up		chr20
A_11_P098666	4.8091803	2.265791	4.8091803	up	C4BPA	chr7
A_11_P0000032909	4.128217	2.0455189	4.128217	up	LOC489748	chr5
A_11_P129001	-4.8757915	-2.2856364	4.8757915	down		chr27
A_11_P190123	-306.9216	-8.261726	306.9216	down	ACTA1	chr4
A_11_P0000035026	-6.4031973	-2.6787925	6.4031973	down		chr9
A_11_P000001298	-3.580683	-1.8402348	3.580683	down		chr28
A_11_P0000017005	-4.1955004	-2.068843	4.1955004	down		chr6
A_11_P190853	11.103931	3.4729986	11.103931	up		chr18
A_11_P0000019712	3.0271957	1.5979819	3.0271957	up	KLRA1	chr27
A_11_P052526	-3.482	-1.7999163	3.482	down	QPCT	chr17
A_11_P154608	5.9174976	2.5649872	5.9174976	up		chr20
A_11_P120681	-3.0631104	-1.6149974	3.0631104	down		

A_11_P125761	-3.205583	-1.6805868	3.205583	down		chr21
A_11_P0000020149	-3.6207426	-1.8562856	3.6207426	down	SLC5A1	chr26
A_11_P0000026702	13.659145	3.7717953	13.659145	up	RASSF6	chr13
A_11_P218663	5.288434	2.4028406	5.288434	up	BPI	chr24
A_11_P0000031113	-7.0045433	-2.808291	7.0045433	down	TGM5	chr30
A_11_P000008413	8.757493	3.130518	8.757493	up		chr1
A_11_P097646	-3.204059	-1.6799006	3.204059	down		chr6
A_11_P085686	-3.3173254	-1.7300205	3.3173254	down	ELOVL1	chr15
A_11_P064971	-5.12874	-2.3586044	5.12874	down	FETUB	chr34
A_11_P000002439	-3.1928658	-1.6748519	3.1928658	down	CREB5	chr14
A_11_P050991	10.143258	3.3424492	10.143258	up	CEACAM23	chr1
A_11_P0000010694	-3.0409713	-1.6045322	3.0409713	down		chr6
A_11_P097076	-5.310216	-2.4087706	5.310216	down		chr6
A_11_P184733	10.594355	3.4052238	10.594355	up	C1QC	chr2
A_11_P0000023699	-3.0783007	-1.6221342	3.0783007	down	ABI3BP	chr33
A_11_P181433	25.88183	4.6938677	25.88183	up	VIL1	chr37
A_11_P0000033981	-10.509822	-3.3936663	10.509822	down	KRT25	chr9
A_11_P109361	-5.624822	-2.4918075	5.624822	down	ADAMTSL3	chr3
A_11_P0000021165	6.8594747	2.778098	6.8594747	up	MYL7	chrUn_JH373316
A_11_P072816	-4.7361484	-2.2437143	4.7361484	down	SLC26A4	chr18
A_11_P0000031495	-3.1801138	-1.6690784	3.1801138	down	MUC4	chr33
A_11_P076936	111.69483	6.8034186	111.69483	up	C3	chr20
A_11_P127291	-7.7001	-2.9448771	7.7001	down		chr10
A_11_P0000038822	-3.7305229	-1.8993778	3.7305229	down		chr10
A_11_P000001058	11.631641	3.5399828	11.631641	up	LOC609288	chr18
A_11_P0000020370	-3.9015758	-1.964057	3.9015758	down	APOL5	chr10
A_11_P0000019508	4.917152	2.297823	4.917152	up	ICAM1	chr20
A_11_P0000034630	5.653168	2.4990597	5.653168	up		chrX

A_11_P136836	-218.69893	-7.7728024	218.69893	down	ACTA1	chr4
A_11_P0000025622	-3.1925387	-1.6747041	3.1925387	down	ASB9	chrX
A_11_P0000020211	4.509433	2.172946	4.509433	up	SLC11A1	chr37
A_11_P0000020727	-3.2256682	-1.6895981	3.2256682	down	FAM135A	chr12
A_11_P0000025508	3.4186065	1.7734084	3.4186065	up	AK8	chr9
A_11_P107976	7.1759706	2.843174	7.1759706	up	CDH13	chr5
A_11_P0000019808	-4.673485	-2.2244987	4.673485	down	CES1	chr2
A_11_P0000021114	-3.5951397	-1.8460479	3.5951397	down		chr15
A_11_P172678	3.0107236	1.5901103	3.0107236	up		chr7
A_11_P120536	7.0943084	2.826662	7.0943084	up		chr15
A_11_P200833	4.158775	2.0561585	4.158775	up		chr5
A_11_P183733	-10.412153	-3.3801966	10.412153	down	HSD17B14	chr1
A_11_P104036	4.267684	2.0934534	4.267684	up	RHOU	chr4
A_11_P0000033970	-10.620425	-3.4087696	10.620425	down	KRT35	chr9
A_11_P081046	-3.5966659	-1.8466601	3.5966659	down	MAPT	chr9
A_11_P000008526	3.2837732	1.7153544	3.2837732	up		chr1
A_11_P166993	6.2034526	2.6330714	6.2034526	up		chr15
A_11_P181438	3.3924134	1.7623119	3.3924134	up	PAX6	
A_11_P00000285	3.2342248	1.6934199	3.2342248	up	CHRDL2	chr21
A_11_P069706	3.428672	1.7776499	3.428672	up	UBE2D3	chr32
A_11_P0000022561	-5.2589836	-2.394784	5.2589836	down		chrUn_JH373528
A_11_P123486	33.036064	5.04597	33.036064	up		chr12
A_11_P053541	3.6170883	1.8548288	3.6170883	up	BPI	chr24
A_11_P123306	4.215094	2.0755649	4.215094	up		chr30
A_11_P153108	3.180857	1.6694155	3.180857	up	APOE	chr1
A_11_P0000025428	-4.543219	-2.1837149	4.543219	down	LOC480601	chr9
A_11_P0000014995	-3.809898	-1.9297523	3.809898	down		chr5
A_11_P0000028130	-7.1981096	-2.847618	7.1981096	down		chr1

A_11_P0000041951	3.530594	1.819911	3.530594	up		chr9
A_11_P0000021954	-80.0981	-6.323696	80.0981	down	CKM	chr1
A_11_P111316	5.0565414	2.338151	5.0565414	up	NTRK2	chr1
A_11_P0000028602	-3.0029204	-1.5863662	3.0029204	down	FBXO27	chr1
A_11_P122596	-6.885465	-2.783554	6.885465	down	LMOD2	chr14
A_11_P0000014853	3.112202	1.6379356	3.112202	up		chr11
A_11_P0000019730	4.2594585	2.09067	4.2594585	up	GNB3	chr27
A_11_P0000021155	-3.3711784	-1.753253	3.3711784	down	FNIP2	chr15
A_11_P169683	-3.657658	-1.8709202	3.657658	down	CHL1	chr20
A_11_P112481	3.006248	1.587964	3.006248	up	LOC484343	chr1
A_11_P176058	-3.4880729	-1.8024302	3.4880729	down	SERPINB5	chr1
A_11_P0000034802	-3.1692305	-1.6641326	3.1692305	down	CHRDL1	chrX
A_11_P0000018029	-3.0203786	-1.5947294	3.0203786	down		chr27
A_11_P102366	5.5348215	2.4685369	5.5348215	up	PPP2R2B	chr2
A_11_P054946	3.8462229	1.9434423	3.8462229	up	FCER1G	chr38
A_11_P176748	-3.1523294	-1.6564183	3.1523294	down		chr23
A_11_P102396	-6.023458	-2.590592	6.023458	down	C2H5orf46	chr2
A_11_P0000019976	-3.9310703	-1.9749222	3.9310703	down	KLK1	chr1
A_11_P0000016394	14.744006	3.8820567	14.744006	up		chr28
A_11_P0000038750	-7.2520766	-2.8583941	7.2520766	down		chr8
A_11_P0000038820	-6.198667	-2.631958	6.198667	down		chr11
A_11_P113191	-3.3315303	-1.7361851	3.3315303	down	PINLYP	chr1
A_11_P0000010651	3.767535	1.913621	3.767535	up		chr24
A_11_P125391	-13.881069	-3.7950468	13.881069	down	LOC100683419	chr17
A_11_P0000038858	-4.312465	-2.1085129	4.312465	down		chr26
A_11_P0000028907	-5.1679454	-2.3695908	5.1679454	down	EPHX3	chr20
A_11_P0000033275	4.239189	2.0837884	4.239189	up	TGFBR3	chr6
A_11_P143073	34.574654	5.111643	34.574654	up	APOC1	chr1

A_11_P155918	-4.4508634	-2.1540852	4.4508634	down		chr9
A_11_P0000013960	3.8018498	1.9267015	3.8018498	up	C3H4orf48	chr3
A_11_P0000040386	28.253689	4.8203673	28.253689	up		chr23
A_11_P0000015369	-4.0997877	-2.0355492	4.0997877	down	NMT2	chr2
A_11_P117041	4.816434	2.2679653	4.816434	up		chr17
A_11_P0000020204	14.317838	3.8397417	14.317838	up	PLP1	chrX
A_11_P0000040377	-4.4973345	-2.1690702	4.4973345	down		chr3
A_11_P000005569	-6.164671	-2.624024	6.164671	down		chr37
A_11_P0000024014	-3.0771055	-1.6215739	3.0771055	down	WDFY1	chr37
A_11_P0000020109	-5.5387616	-2.4695635	5.5387616	down	CCL21	chr11
A_11_P143758	3.9293194	1.9742794	3.9293194	up	SAA1	chr21
A_11_P169198	4.6927714	2.2304401	4.6927714	up		chr10
A_11_P164213	3.4017067	1.7662587	3.4017067	up		chr8
A_11_P0000026054	-4.87785	-2.2862453	4.87785	down		chr10
A_11_P062681	-3.440528	-1.78263	3.440528	down	PAQR5	chr30
A_11_P174863	3.0826688	1.6241798	3.0826688	up		chr6
A_11_P116486	-4.1783447	-2.0629315	4.1783447	down	KHDRBS3	chr13
A_11_P0000024871	4.430734	2.1475458	4.430734	up	BCAR3	chr6
A_11_P070001	-5.035582	-2.3321586	5.035582	down	MEDAG	chr25
A_11_P088751	-5.205996	-2.3801742	5.205996	down	MYRFL	chr10
A_11_P00000760	-7.556781	-2.9177718	7.556781	down		chr37
A_11_P082721	4.3683243	2.12708	4.3683243	up	ABHD15	chr9
A_11_P0000031268	-82.184074	-6.360787	82.184074	down	KRTAP11-1	chr31
A_11_P0000026057	5.203742	2.3795495	5.203742	up		chr10
A_11_P0000029552	-4.276481	-2.096424	4.276481	down	ESYT3	chr23
A_11_P058851	-3.0956674	-1.6302505	3.0956674	down	FBXO45	chr33
A_11_P102986	-3.8521895	-1.9456787	3.8521895	down	TMEM54	chr2
A_11_P0000024032	-4.5596495	-2.188923	4.5596495	down	CFH	chr38

A_11_P050916	-4.6213546	-2.2083158	4.6213546	down	ANGPT2	chr16
A_11_P073931	8.999501	3.169845	8.999501	up	IGF2	chr18
A_11_P111001	-3.2103949	-1.6827507	3.2103949	down	FAM162B	chr1
A_11_P0000013744	3.1345248	1.6482468	3.1345248	up		chr11
A_11_P066811	-3.5753884	-1.8381	3.5753884	down	SPTLC3	chr24
A_11_P0000031274	3.3672786	1.7515831	3.3672786	up		chr31
A_11_P0000014985	-3.3632154	-1.7498412	3.3632154	down	RAPGEF4	chr36
A_11_P123291	4.5838637	2.1965642	4.5838637	up	C4BPA	chr7
A_11_P051256	3.366722	1.7513447	3.366722	up	LGALS9	chr9
A_11_P0000023928	3.9866087	1.995162	3.9866087	up	FRZB	chr36
A_11_P0000020738	-3.9124076	-1.9680567	3.9124076	down	SH3BGRL2	chr12
A_11_P163053	-3.3420496	-1.7407331	3.3420496	down		chr25
A_11_P121006	4.50063	2.170127	4.50063	up		chr18
A_11_P102926	3.26479	1.7069902	3.26479	up		chr2
A_11_P0000039403	15.002993	3.9071784	15.002993	up		chr10
A_11_P159268	-3.307435	-1.7257128	3.307435	down		chr5
A_11_P084951	-3.038542	-1.6033792	3.038542	down		chr15
A_11_P051101	4.177574	2.0626655	4.177574	up	KLRD1	chr27
A_11_P076996	-6.0989666	-2.6085649	6.0989666	down	ACER1	chr20
A_11_P0000027695	4.8497686	2.277916	4.8497686	up	B4GALNT4	chr18
A_11_P088026	-3.3590462	-1.7480516	3.3590462	down	HJV	chr17
A_11_P0000018738	3.3104272	1.7270174	3.3104272	up		chr12
A_11_P0000031372	-10.031362	-3.3264456	10.031362	down	MMRN1	chr32
A_11_P084936	-3.321209	-1.7317085	3.321209	down	EREG	chr13
A_11_P0000016871	6.4745884	2.6947885	6.4745884	up	TCEAL5	chrX
A_11_P140476	3.2878642	1.7171507	3.2878642	up	IFGGB1	chr11
A_11_P0000027369	-5.1082172	-2.35282	5.1082172	down	GALNT14	chr17
A_11_P0000011111	-3.949885	-1.9818106	3.949885	down		chr37

A_11_P112546	-4.3357124	-2.116269	4.3357124	down	KLK10	chr1
A_11_P088276	-12.101529	-3.5971174	12.101529	down	TCHH	chr17
A_11_P0000040883	-4.9264894	-2.30056	4.9264894	down		chr3
A_11_P199318	-3.5545657	-1.8296733	3.5545657	down		chr5
A_11_P050701	3.6588356	1.8713846	3.6588356	up	OAS2	chr26
A_11_P0000025225	3.7173812	1.8942866	3.7173812	up	PLEKHH1	chr8
A_11_P196633	3.4826722	1.8001947	3.4826722	up	COL12A1	chr12
A_11_P153288	3.7136025	1.8928194	3.7136025	up		chr22
A_11_P0000028716	-4.9134545	-2.2967377	4.9134545	down	TRH	chr20
A_11_P190213	3.7127779	1.892499	3.7127779	up		chr20
A_11_P0000019743	-3.8708158	-1.9526377	3.8708158	down	RHBG	chr7
A_11_P0000040346	6.3965573	2.6772957	6.3965573	up		chr10
A_11_P154853	8.376099	3.0662785	8.376099	up		chr38
A_11_P0000016622	-3.1274672	-1.6449947	3.1274672	down		chr3
A_11_P050886	4.1246943	2.0442872	4.1246943	up	MMP3	chr5
A_11_P0000016966	-3.6769633	-1.8785148	3.6769633	down		chr36
A_11_P165173	18.163834	4.1829967	18.163834	up	CIDEA	chr7
A_11_P175003	27.944584	4.804497	27.944584	up		chr7
A_11_P0000033360	-4.522242	-2.1770382	4.522242	down	YOD1	chr7
A_11_P189053	-63.10131	-5.979598	63.10131	down		chr24
A_11_P114341	8.92452	3.1577744	8.92452	up	DMD	chrX
A_11_P0000011863	3.5775907	1.8389883	3.5775907	up		chr33
A_11_P099226	-5.4805236	-2.4543138	5.4805236	down		chr7
A_11_P057301	-3.043954	-1.6059465	3.043954	down	ITPRID2	chr36
A_11_P0000016349	-3.1995676	-1.677877	3.1995676	down	SLC13A2	chr9
A_11_P000005639	5.036676	2.3324718	5.036676	up		chr15
A_11_P056906	-5.322868	-2.4122038	5.322868	down	DAPL1	chr36
A_11_P0000026535	4.2128267	2.0747886	4.2128267	up	OSR2	chr13

A_11_P0000019718	-4.4068074	-2.1397338	4.4068074	down	TFF1	chr31
A_11_P0000019869	-25.463228	-4.6703434	25.463228	down	MT4	chr2
A_11_P0000025605	3.316522	1.729671	3.316522	up	FN3K	chrUn_JH373243
A_11_P0000033972	-13.01028	-3.70158	13.01028	down	KRT31	chr9
A_11_P210773	-4.4897184	-2.166625	4.4897184	down		chr1
A_11_P0000016551	-3.9176352	-1.9699831	3.9176352	down		chr33
A_11_P0000023452	3.5104442	1.8116536	3.5104442	up	SCG5	chr30
A_11_P093771	-8.804158	-3.138185	8.804158	down	SERPINA9	chr8
A_11_P0000018275	4.841436	2.275435	4.841436	up		chr34
A_11_P126391	17.658607	4.1422997	17.658607	up	VIT	chr17
A_11_P194538	9.236208	3.2073007	9.236208	up	STC1	chr25
A_11_P0000039633	-10.097679	-3.3359518	10.097679	down		chr5
A_11_P138311	3.5414562	1.8243427	3.5414562	up		chr24
A_11_P00000977	8.401038	3.0705676	8.401038	up	CDH13	chr5
A_11_P155233	3.437192	1.7812304	3.437192	up		chr14
A_11_P0000016126	4.1916943	2.0675335	4.1916943	up		chr20
A_11_P073018	3.1929681	1.6748981	3.1929681	up		chr18
A_11_P0000024108	10.379954	3.3757281	10.379954	up	AP3B2	chr3
A_11_P0000039747	5.792447	2.534173	5.792447	up		chr3
A_11_P0000031685	-3.1820042	-1.6699357	3.1820042	down		chr35
A_11_P107731	5.652664	2.498931	5.652664	up	LOC489640	chr5
A_11_P198223	-6.9270296	-2.7922368	6.9270296	down	POSTN	chr25
A_11_P000006271	5.808755	2.538229	5.808755	up		chr4
A_11_P0000016684	-5.078582	-2.3444257	5.078582	down		chrX
A_11_P0000014179	-20.567673	-4.3623066	20.567673	down		chrUn_JH373728
A_11_P0000026598	-5.3809547	-2.4278622	5.3809547	down	SLURP1	chr13
A_11_P0000025321	3.8365405	1.939806	3.8365405	up	LIMD2	chr9
A_11_P182663	-3.7476082	-1.9059701	3.7476082	down	RBP4	chr28

A_11_P187508	3.8995984	1.9633255	3.8995984	up	RPSA	chr23
A_11_P143123	-29.158907	-4.8658648	29.158907	down		chr24
A_11_P160483	-482.80746	-8.915304	482.80746	down	MYL1	chr37
A_11_P0000021233	3.9639976	1.9869561	3.9639976	up	FGFR1	chr16
A_11_P145193	-3.8686619	-1.9518347	3.8686619	down	DUSP14	chr9
A_11_P0000032500	-3.4906447	-1.8034935	3.4906447	down	BARX2	chr5
A_11_P0000032082	3.657044	1.870678	3.657044	up	BCL2A1	chr3
A_11_P084836	-3.2296646	-1.6913843	3.2296646	down		chr13
A_11_P0000020102	3.6840968	1.8813109	3.6840968	up	CCL3	chr9
A_11_P197508	-3.2664416	-1.7077198	3.2664416	down		chr11
A_11_P0000027663	3.1677716	1.6634684	3.1677716	up	SLC38A10	chr9
A_11_P00000112	-3.157101	-1.6586003	3.157101	down	KANK1	chr1
A_11_P200538	-3.1436074	-1.652421	3.1436074	down	EMP1	chr27
A_11_P069326	3.427487	1.7771511	3.427487	up	ZNF215	chr21
A_11_P0000040384	-8.234599	-3.0416985	8.234599	down		chr28
A_11_P0000016647	-4.03006	-2.0108013	4.03006	down		chrX
A_11_P051981	3.2707682	1.7096295	3.2707682	up	CP	chr23
A_11_P0000039717	-3.0987763	-1.6316986	3.0987763	down		chr36
A_11_P150868	-6.8615584	-2.7785363	6.8615584	down		chr2
A_11_P000004647	-13.096104	-3.7110658	13.096104	down		
A_11_P131441	-8.250643	-3.0445065	8.250643	down	SNX29	chr6
A_11_P176223	9.493723	3.246974	9.493723	up		chr6
A_11_P0000021904	3.169812	1.6643972	3.169812	up	TMEM190	chr1
A_11_P0000020374	-4.1446285	-2.0512428	4.1446285	down	BPIFC	chr10
A_11_P0000023718	-6.464959	-2.6926413	6.464959	down	CCDC80	chr33
A_11_P181593	3.3572083	1.747262	3.3572083	up	LOC607937	chr8
A_11_P157538	-9.874153	-3.303657	9.874153	down	EHD2	chr1
A_11_P053163	-3.4311128	-1.7786765	3.4311128	down	VNN1	chr1

A_11_P0000026751	-11.937425	-3.5774198	11.937425	down	CPA4	chr14
A_11_P199403	-58.018356	-5.8584375	58.018356	down		chr24
A_11_P0000034261	-8.094787	-3.016993	8.094787	down	TTC16	chr9
A_11_P081236	-3.9559717	-1.9840322	3.9559717	down	SLC16A6	chr9
A_11_P0000033039	-3.6293032	-1.8596926	3.6293032	down	TMEM184A	chr6
A_11_P0000016413	-3.7075698	-1.8904738	3.7075698	down	GNMT	chr12
A_11_P0000021261	-3.4666631	-1.7935476	3.4666631	down	FGL1	chr16
A_11_P079491	-4.3066597	-2.1065693	4.3066597	down	HOXA6	chr14
A_11_P148198	9.155062	3.1945696	9.155062	up		chr4
A_11_P153818	3.3650923	1.7506461	3.3650923	up	CASK	chrX
A_11_P145218	-24.787777	-4.631557	24.787777	down		chr6
A_11_P153483	4.713377	2.236761	4.713377	up		chr18
A_11_P185718	4.520798	2.1765776	4.520798	up		chr33
A_11_P149703	-3.2381973	-1.6951909	3.2381973	down		chr2
A_11_P0000018507	-4.649536	-2.2170868	4.649536	down		chr20
A_11_P0000024240	5.014421	2.3260832	5.014421	up	RHOU	chr4
A_11_P143313	-4.913055	-2.2966204	4.913055	down	ACTA1	chr4
A_11_P0000020676	-3.2168717	-1.6856585	3.2168717	down		chr12
A_11_P0000015138	-4.7283826	-2.2413468	4.7283826	down		chr24
A_11_P0000030654	-3.4544156	-1.7884417	3.4544156	down	CA8	chr29
A_11_P0000026523	-4.1241584	-2.0440998	4.1241584	down	RFPL4B	chr12
A_11_P106986	3.951248	1.9823084	3.951248	up	ALDH3A2	chr5
A_11_P0000021306	-4.114934	-2.0408692	4.114934	down	CYRIA	chr17
A_11_P0000021115	-7.870346	-2.976427	7.870346	down	HAL	chr15
A_11_P127736	-6.61922	-2.7266612	6.61922	down	MYOZ1	chr4
A_11_P088306	-27.811907	-4.797631	27.811907	down	PRR9	chr17
A_11_P144693	-5.1731267	-2.3710365	5.1731267	down	ACTA1	chr4
A_11_P0000040479	-5.1447554	-2.3631024	5.1447554	down		chr13

A_11_P066316	3.5009823	1.8077598	3.5009823	up	RIMKLB	chr27
A_11_P201098	-8.177176	-3.0316029	8.177176	down	TPM1	chr30
A_11_P204798	3.6994994	1.88733	3.6994994	up		chr9
A_11_P090946	-5.436086	-2.4425683	5.436086	down	SPDEF	chr12
A_11_P0000037144	-3.3282583	-1.7347674	3.3282583	down	RASD2	chr10
A_11_P000004032	-5.3748116	-2.4262142	5.3748116	down		chr32
A_11_P0000014216	-4.037685	-2.0135283	4.037685	down		chr16
A_11_P088376	3.0460513	1.6069403	3.0460513	up	C17H1orf162	chr17
A_11_P0000041364	5.2590756	2.3948092	5.2590756	up		chr18
A_11_P0000024275	-10.50549	-3.3930717	10.50549	down	MYOZ1	chr4
A_11_P148283	4.643864	2.2153258	4.643864	up		chr1
A_11_P088516	-3.5970747	-1.8468242	3.5970747	down	RDH16	chr10
A_11_P0000017318	-3.7475362	-1.9059424	3.7475362	down		chrX
A_11_P190678	5.567843	2.4771185	5.567843	up	FRMD4A	chr2
A_11_P0000030235	3.350629	1.744432	3.350629	up	LIPA	chr26
A_11_P0000040953	7.463068	2.8997688	7.463068	up		chr12
A_11_P181188	-59.00588	-5.8827868	59.00588	down		chr24
A_11_P051156	-6.183075	-2.6283245	6.183075	down	PTGS1	chr9
A_11_P206293	-192.7376	-7.590494	192.7376	down	ACTA1	chr4
A_11_P0000040658	-3.3137233	-1.7284532	3.3137233	down	MYPN	chr4
A_11_P179953	-222.71326	-7.7990437	222.71326	down	ACTA1	chr4
A_11_P0000034543	-12.736475	-3.6708941	12.736475	down	ARSF	chrX
A_11_P0000019918	7.5828013	2.922731	7.5828013	up	CXCL8	chr13
A_11_P0000027938	-4.9872284	-2.3182383	4.9872284	down	CD248	chr18
A_11_P0000023651	-3.8346224	-1.9390845	3.8346224	down	HPGDS	chr32
A_11_P193673	-5.1840744	-2.3740864	5.1840744	down	OTUD5	chrX
A_11_P051191	5.2378445	2.3889732	5.2378445	up	SAA1	chr21
A_11_P0000039106	4.811492	2.2664843	4.811492	up		chr18

A_11_P053576	-3.1645267	-1.6619897	3.1645267	down	EDN1	chr35
A_11_P0000026942	-5.4455576	-2.4450798	5.4455576	down	GJB4	chr15
A_11_P0000023062	-3.712503	-1.8923922	3.712503	down	RBP4	chr28
A_11_P0000030687	-3.0892076	-1.6272368	3.0892076	down	PI15	chr29
A_11_P0000039652	-3.9108586	-1.9674854	3.9108586	down		chr30
A_11_P0000019855	3.2398095	1.695909	3.2398095	up	MX1	chr31
A_11_P0000021299	-3.0663185	-1.6165075	3.0663185	down		chr17
A_11_P217273	-13.413853	-3.7456517	13.413853	down	LOC100683419	chr17
A_11_P0000032694	-3.1857345	-1.6716261	3.1857345	down	MYH8	chr5
A_11_P095756	-3.331396	-1.7361269	3.331396	down		chr11
A_11_P0000040689	-7.6877103	-2.942554	7.6877103	down		chr26
A_11_P0000026277	-3.6334453	-1.8613381	3.6334453	down	SLC31A2	chr11
A_11_P00000876	9.203157	3.202129	9.203157	up		chrX
A_11_P153778	-4.214926	-2.0755072	4.214926	down		chr21
A_11_P175733	5.9292088	2.5678396	5.9292088	up		chr8
A_11_P109826	3.9096322	1.9670329	3.9096322	up	TLR1	chr3
A_11_P155108	-3.4019866	-1.7663774	3.4019866	down	WFDC5	chr24
A_11_P069501	3.5937605	1.8454943	3.5937605	up	ARNTL	chr21
A_11_P0000017224	3.1702583	1.6646004	3.1702583	up		chr11
A_11_P0000033760	-3.6579676	-1.8710423	3.6579676	down	RDH12	chr8
A_11_P115341	4.5993648	2.2014346	4.5993648	up	GPRASP2	chrX
A_11_P0000019865	3.161404	1.6605654	3.161404	up	CD86	chr33
A_11_P0000028608	-4.9288282	-2.3012447	4.9288282	down		chr1
A_11_P0000032925	3.4129007	1.7709985	3.4129007	up	HSF4	chr5
A_11_P058961	5.6958117	2.5099015	5.6958117	up	CXCL10	chr32
A_11_P143643	3.197896	1.6771231	3.197896	up		chr17
A_11_P0000031865	-4.1704364	-2.0601983	4.1704364	down	MAP2	chr37
A_11_P168338	-4.683663	-2.2276373	4.683663	down		chr1

A_11_P0000018380	5.174818	2.3715081	5.174818	up		chr1
A_11_P0000027732	3.4731772	1.7962561	3.4731772	up	PAX6	
A_11_P0000039074	3.4203606	1.7741485	3.4203606	up		chr30
A_11_P086661	-3.6566691	-1.8705301	3.6566691	down	ATP6V1C2	chr17
A_11_P0000040812	5.905544	2.56207	5.905544	up	BMX	chrX
A_11_P0000032406	3.0031664	1.5864844	3.0031664	up		chr4
A_11_P195190	4.936592	2.3035154	4.936592	up		chr18
A_11_P0000039621	-3.2765527	-1.7121787	3.2765527	down	KLK4	chr1
A_11_P0000017078	3.6244004	1.8577423	3.6244004	up		chr6
A_11_P0000016656	3.607675	1.8510695	3.607675	up	FXYD6	chr5
A_11_P161123	-3.229197	-1.6911755	3.229197	down	OSBPL1A	chr7
A_11_P195453	-16.461966	-4.0410647	16.461966	down	CACNA1S	chr7
A_11_P111146	-7.58018	-2.9222322	7.58018	down		chr1
A_11_P138231	5.735804	2.5199957	5.735804	up	MGST1	chr27
A_11_P0000027088	25.211119	4.655988	25.211119	up	TMEM144	chr15
A_11_P166943	3.2399316	1.6959634	3.2399316	up		chr8
A_11_P0000037025	3.1157238	1.6395674	3.1157238	up		chr13
A_11_P000003923	16.441471	4.0392675	16.441471	up		chr5
A_11_P090746	9.162417	3.1957283	9.162417	up	C2	chr12
A_11_P104211	3.211973	1.6834598	3.211973	up	FAM241B	chr4
A_11_P146158	-6.249384	-2.643714	6.249384	down	DCN	chr15
A_11_P137406	-8.872101	-3.1492758	8.872101	down	TCAP	chr9
A_11_P0000040699	-4.483898	-2.1647534	4.483898	down		chr25
A_11_P0000026287	-3.2965224	-1.7209449	3.2965224	down	KIF12	chr11
A_11_P0000019862	3.4257777	1.7764316	3.4257777	up	CD38	chr3
A_11_P198953	3.3599522	1.7484407	3.3599522	up	PALM2AKAP2	chr11
A_11_P105661	8.275639	3.0488706	8.275639	up	ROBO3	chr5
A_11_P0000016844	-3.9041843	-1.9650211	3.9041843	down		chr25

A_11_P0000029534	-4.721519	-2.2392511	4.721519	down	ACP3	chr23
A_11_P0000032628	-5.107439	-2.3526	5.107439	down	MMP27	chr5
A_11_P0000034008	3.2863789	1.7164989	3.2863789	up	CISD3	chr9
A_11_P0000025427	9.758464	3.286654	9.758464	up	LOC480600	chr9
A_11_P063241	6.370292	2.6713595	6.370292	up	ANKRD2	chr28
A_11_P0000030994	5.702469	2.5115867	5.702469	up	EPHB2	chr2
A_11_P0000027189	-6.728955	-2.7503824	6.728955	down		chr16
A_11_P0000018701	-6.2917295	-2.6534567	6.2917295	down		chr30
A_11_P150478	-5.7188993	-2.5157375	5.7188993	down		chr18
A_11_P0000027998	4.664032	2.2215776	4.664032	up	SLC3A2	chr18
A_11_P051166	105.86822	6.7261257	105.86822	up	PPARG	chr20
A_11_P0000024409	3.2029884	1.6794186	3.2029884	up	CD3D	chr5
A_11_P0000038794	414.09366	8.693813	414.09366	up		chr34
A_11_P137761	-4.710234	-2.2357988	4.710234	down	DAAM1	chr8
A_11_P0000019937	22.214674	4.473441	22.214674	up	MMP9	chr24
A_11_P0000022297	-4.33677	-2.116621	4.33677	down	HPX	chr21
A_11_P103781	16.014454	4.0013027	16.014454	up	FBXO2	chr2
A_11_P00000805	-14.273673	-3.8352847	14.273673	down	TCAP	chr9
A_11_P073151	-3.8588033	-1.9481535	3.8588033	down	ELF5	chr18
A_11_P151618	-111.94353	-6.8066273	111.94353	down		chr4
A_11_P212963	3.6857104	1.8819427	3.6857104	up	MYEF2	chr30
A_11_P063271	-4.5393467	-2.1824846	4.5393467	down	CRTAC1	chr28
A_11_P054136	4.5605083	2.1891947	4.5605083	up	TGFB2	chr38
A_11_P099616	-3.9850357	-1.9945927	3.9850357	down	TMEM79	chr7
A_11_P0000028664	3.6802619	1.8798084	3.6802619	up	HPN	chr1
A_11_P000004074	-3.707925	-1.8906121	3.707925	down	TAL1	chr15
A_11_P0000015868	3.1734977	1.6660738	3.1734977	up		chr6
A_11_P081626	-81.16068	-6.342709	81.16068	down	KRT34	chr9

A_11_P0000015292	19.81603	4.308596	19.81603	up		chr27
A_11_P000004855	3.1807811	1.6693811	3.1807811	up	COL12A1	chr12
A_11_P0000023709	3.1727028	1.6657124	3.1727028	up	TRAT1	chr33
A_11_P178938	-3.584979	-1.8419647	3.584979	down		chr30
A_11_P0000025419	-5.909234	-2.562971	5.909234	down	CA4	chr9
A_11_P00000209	-3.658398	-1.871212	3.658398	down	KCNS3	chr17
A_11_P0000014550	3.7197657	1.8952117	3.7197657	up	GIMAP8	chr16
A_11_P193453	7.480884	2.9032087	7.480884	up	C1QC	chr2
A_11_P054146	-3.3762476	-1.7554207	3.3762476	down	SCN9A	chr36
A_11_P138566	12.405905	3.632955	12.405905	up	GATM	chr30
A_11_P0000031576	-5.1032696	-2.3514218	5.1032696	down	LIPH	chr34
A_11_P0000030406	-3.4399345	-1.782381	3.4399345	down	EMP1	chr27
A_11_P066126	-3.1798878	-1.6689758	3.1798878	down	PDE6H	chr27
A_11_P093161	-4.2480693	-2.0868073	4.2480693	down	HSPA2	chr8
A_11_P0000024758	-10.560461	-3.400601	10.560461	down	CRYM	chr6
A_11_P0000013056	-5.6064672	-2.487092	5.6064672	down	CCL21	chr11
A_11_P0000030796	-8.977069	-3.1662445	8.977069	down		chr2
A_11_P051306	-4.147382	-2.0522008	4.147382	down	HMGN2	chr2
A_11_P0000034042	4.23866	2.0836082	4.23866	up	ITGA3	chr9
A_11_P166668	6.089385	2.6062965	6.089385	up	MGST1	chr27
A_11_P0000029610	8.8839	3.1511931	8.8839	up	BFSP1	chr24
A_11_P221303	-3.8260074	-1.9358397	3.8260074	down	GPX3	chr4
A_11_P0000029872	-4.015116	-2.0054417	4.015116	down	AADAT	chr25
A_11_P0000016385	3.1279614	1.6452227	3.1279614	up	TECPR1	chr6
A_11_P124561	-4.753721	-2.2490573	4.753721	down		chr17
A_11_P093341	4.90267	2.2935677	4.90267	up	SIPA1L1	chr8
A_11_P00000688	-3.2378817	-1.6950502	3.2378817	down	LTF	chr20
A_11_P076931	121.35141	6.923047	121.35141	up	C3	chr20

A_11_P060816	3.206416	1.6809616	3.206416	up		chr26
A_11_P136716	-198.68745	-7.634357	198.68745	down	LOC476825	chr21
A_11_P182203	4.528524	2.179041	4.528524	up	FXYD6	chr5
A_11_P0000031755	4.332137	2.115079	4.332137	up	PLA2R1	chr36
A_11_P077131	4.818225	2.2685018	4.818225	up	MATK	chr20
A_11_P110206	-3.0882561	-1.6267924	3.0882561	down		chr1
A_11_P201093	-3.515401	-1.8136892	3.515401	down		chr20
A_11_P0000026663	-10.999306	-3.4593406	10.999306	down	CWH43	chr13
A_11_P0000033980	-7.36898	-2.881465	7.36898	down	KRT27	chr9
A_11_P206608	4.224753	2.078867	4.224753	up		chr5
A_11_P0000015228	10.734679	3.4242072	10.734679	up		chr3
A_11_P053921	15.979373	3.998139	15.979373	up	EDNRA	chr15
A_11_P0000030230	-5.2835507	-2.4015079	5.2835507	down		chr26
A_11_P081681	-8.043687	-3.0078568	8.043687	down	KRT28	chr9
A_11_P0000024881	-4.016985	-2.006113	4.016985	down	KIAA1107	chr6
A_11_P0000034855	3.5031652	1.8086591	3.5031652	up	GPC4	chrX
A_11_P0000010441	-9.183802	-3.1990914	9.183802	down		
A_11_P0000017798	-4.1739373	-2.061409	4.1739373	down		chr25
A_11_P0000023104	18.89473	4.239912	18.89473	up	ACSL5	chr28
A_11_P0000017264	3.4174068	1.772902	3.4174068	up		chr14
A_11_P0000015406	-3.4357257	-1.7806149	3.4357257	down		chr37
A_11_P099836	-3.2427547	-1.6972198	3.2427547	down	S100A14	chr7
A_11_P157263	3.1703327	1.6646342	3.1703327	up	ART3	chr32
A_11_P0000030995	9.725939	3.2818375	9.725939	up	C1QB	chr2
A_11_P0000030897	-4.202792	-2.0713482	4.202792	down	CAPNS2	chr2
A_11_P000002531	-39.601852	-5.307496	39.601852	down		chr36
A_11_P178478	-11.195111	-3.484797	11.195111	down	TCAP	chr9
A_11_P179373	3.903467	1.964756	3.903467	up		chr3

A_11_P052941	3.0656831	1.6162086	3.0656831	up	LOC100683387	chrUn_JH373254
A_11_P149958	6.6905994	2.7421355	6.6905994	up	SLC6A1	chr20
A_11_P067036	-24.271454	-4.6011887	24.271454	down		chr24
A_11_P0000028733	4.3340163	2.1157045	4.3340163	up		chr20
A_11_P078486	-3.4219923	-1.7748365	3.4219923	down		chr16
A_11_P0000020455	5.426694	2.4400735	5.426694	up	MEIS1	chr10
A_11_P111651	4.0007257	2.0002618	4.0007257	up	CD274	chr1
A_11_P0000028525	4.682971	2.2274241	4.682971	up	CCDC8	chr1
A_11_P0000019827	3.0794344	1.6226654	3.0794344	up	CTLA4	chr37
A_11_P0000017220	5.032027	2.3311396	5.032027	up		chr5
A_11_P0000027957	-8.078771	-3.0141358	8.078771	down	KCNK7	chr18
A_11_P171573	-3.07113	-1.6187696	3.07113	down		chr6
A_11_P075046	6.6688223	2.737432	6.6688223	up		chr20
A_11_P067796	-67.51303	-6.077094	67.51303	down		chr24
A_11_P110666	-3.0024736	-1.5861516	3.0024736	down	PPP1R14C	chr1
A_11_P100511	-4.497882	-2.1692457	4.497882	down	ZNF750	chrUn_JH373243
A_11_P145263	4.0303946	2.010921	4.0303946	up		chrX
A_11_P196218	-11.965099	-3.5807605	11.965099	down	COX6A2	chr6
A_11_P057066	-4.9866743	-2.318078	4.9866743	down	LRP2	chr36
A_11_P081611	-6.322664	-2.6605325	6.322664	down	KRT36	chr9
A_11_P164308	3.630876	1.8603177	3.630876	up	MPP6	chr14
A_11_P076446	-8.054967	-3.0098786	8.054967	down	CASP14	chr20
A_11_P161638	3.5599446	1.8318548	3.5599446	up		chr38
A_11_P0000033561	-9.156042	-3.194724	9.156042	down	DSC1	chr7
A_11_P0000023127	-3.784307	-1.9200292	3.784307	down	PLPP4	chr28
A_11_P0000039311	-3.1924427	-1.6746607	3.1924427	down	GPX3	chr4
A_11_P0000034926	-3.027622	-1.5981851	3.027622	down	L1CAM	chrX
A_11_P0000020825	-9.9120245	-3.3091798	9.9120245	down	CCN3	chr13

A_11_P087376	-12.453129	-3.6384363	12.453129	down	IL37	chr17
A_11_P000001689	-3.8341827	-1.9389191	3.8341827	down		chr32
A_11_P120071	3.4966884	1.8059893	3.4966884	up		chrUn_JH373990
A_11_P0000032858	-3.8566535	-1.9473495	3.8566535	down	GAN	chr5
A_11_P0000015282	17.760666	4.150614	17.760666	up		chrX
A_11_P0000038862	-3.287171	-1.7168465	3.287171	down	MFSD13A	chr28
A_11_P221863	4.1496935	2.0530047	4.1496935	up		chr3
A_11_P119781	-3.5895605	-1.8438072	3.5895605	down	CERS3	chr3
A_11_P086601	-6.2141995	-2.6355686	6.2141995	down	MBOAT2	chr17
A_11_P095621	-3.2163455	-1.6854224	3.2163455	down	LPAR1	chr11
A_11_P0000022860	-3.3800147	-1.7570295	3.3800147	down		chr26
A_11_P0000034122	6.0790086	2.603836	6.0790086	up		chr9
A_11_P219313	-5.7719183	-2.5290508	5.7719183	down	KRT12	chr9
A_11_P071401	3.5715392	1.836546	3.5715392	up		
A_11_P176483	-190.90987	-7.576748	190.90987	down		chr4
A_11_P096681	-13.9251375	-3.7996197	13.9251375	down	MYLPF	chr6
A_11_P0000039619	-3.4331262	-1.7795229	3.4331262	down		chr16
A_11_P0000032631	3.4610407	1.7912059	3.4610407	up	BIRC3	chr5
A_11_P057471	7.0732274	2.8223686	7.0732274	up	STAT4	chr37
A_11_P0000024446	3.3507829	1.7444983	3.3507829	up	MMP12	chr5
A_11_P081606	19.27139	4.2683887	19.27139	up	KRT13	chr9
A_11_P076226	-5.0818005	-2.3453398	5.0818005	down	FCHO1	chr20
A_11_P143498	6.2939844	2.6539736	6.2939844	up		chr18
A_11_P0000039283	3.2871993	1.7168589	3.2871993	up	SERPINH1	chr21
A_11_P165578	-3.641369	-1.864481	3.641369	down		chr24
A_11_P075246	-4.1016636	-2.036209	4.1016636	down	CHL1	chr20
A_11_P0000031353	9.8686495	3.3028526	9.8686495	up	SCD5	chr32
A_11_P193378	-3.566996	-1.8347096	3.566996	down	ENO3	chr5

A_11_P0000023402	6.5765367	2.717328	6.5765367	up	C1QA	chr2
A_11_P081186	3.8494837	1.944665	3.8494837	up	TEX2	chr9
A_11_P0000033381	-3.0732646	-1.619772	3.0732646	down	KCNK2	chr7
A_11_P000001494	-5.1701818	-2.370215	5.1701818	down	COL6A1	chr31
A_11_P0000020203	-4.860755	-2.2811804	4.860755	down	KRT10	chr9
A_11_P154268	4.8646154	2.2823257	4.8646154	up		chr2
A_11_P0000021536	-5.8551574	-2.549708	5.8551574	down	SEMA3D	chr18
A_11_P162378	-6.0146766	-2.5884871	6.0146766	down		chr4
A_11_P184913	-6.0704775	-2.60181	6.0704775	down		chr6
A_11_P0000031137	3.4758806	1.7973785	3.4758806	up	SECISBP2L	chr30
A_11_P0000021670	-3.1201098	-1.6415968	3.1201098	down	HSPA4L	chr19
A_11_P070296	-6.149817	-2.6205435	6.149817	down	LOC477365	chr25
A_11_P0000039664	-4.150157	-2.053166	4.150157	down		chr11
A_11_P092916	-3.6813178	-1.8802223	3.6813178	down		chr8
A_11_P000002492	4.8138013	2.2671766	4.8138013	up		chr1
A_11_P0000022986	6.265147	2.6473484	6.265147	up	MGST1	chr27
A_11_P085606	-3.0670016	-1.6168289	3.0670016	down	PLK3	chr15
A_11_P0000028540	-3.6291711	-1.8596401	3.6291711	down	KLC3	chr1
A_11_P0000031265	-3.5021524	-1.8082418	3.5021524	down		chr31
A_11_P000001054	4.3766646	2.1298318	4.3766646	up		chr18
A_11_P0000031203	3.3756652	1.7551718	3.3756652	up		chr30
A_11_P214643	-7.9852486	-2.9973373	7.9852486	down	MARCHF3	chr11
A_11_P0000033205	-3.8888521	-1.9593444	3.8888521	down	UNKL	chr6
A_11_P0000030706	-4.3713746	-2.128087	4.3713746	down	CA13	chr29
A_11_P091981	-3.0329382	-1.6007161	3.0329382	down	POPDC3	chr12
A_11_P080166	-3.4948282	-1.8052216	3.4948282	down	SCEL	chr22
A_11_P0000033780	-4.0270243	-2.0097141	4.0270243	down	PAPLN	chr8
A_11_P093776	-12.480026	-3.641549	12.480026	down	SERPINA12	chr8

A_11_P0000034315	-5.1149297	-2.3547144	5.1149297	down	RGS19	chr24
A_11_P0000026796	-5.0476546	-2.3356133	5.0476546	down	DLX5	chr14
A_11_P162343	-3.5425918	-1.8248053	3.5425918	down	FGL1	chr16
A_11_P105996	4.265322	2.0926547	4.265322	up	FXYD6	chr5
A_11_P0000021743	-3.510362	-1.8116198	3.510362	down	SERPINB5	chr1
A_11_P000004838	-3.4615543	-1.79142	3.4615543	down		
A_11_P000001138	4.7948146	2.261475	4.7948146	up	LOC609288	chr18
A_11_P084571	-6.06284	-2.5999937	6.06284	down		chr13
A_11_P172003	-4.0083947	-2.0030246	4.0083947	down	PCP4	chr31
A_11_P0000025075	6.6939087	2.7428489	6.6939087	up	ABHD3	chr7
A_11_P196393	-7.619511	-2.9296985	7.619511	down		chr17
A_11_P0000041087	-3.06047	-1.6137533	3.06047	down		chr5
A_11_P0000027726	4.9501076	2.3074598	4.9501076	up	DEPDC7	chr18
A_11_P053036	-6.0418696	-2.594995	6.0418696	down		chr4
A_11_P087381	-5.419703	-2.4382138	5.419703	down	IL36G	chr17
A_11_P0000020638	16.344604	4.0307426	16.344604	up	CFB	chr12
A_11_P0000016630	-9.476888	-3.2444134	9.476888	down		chr3
A_11_P0000021687	-3.0512388	-1.609395	3.0512388	down	DBI	chr19
A_11_P0000034117	-3.469123	-1.7945709	3.469123	down	FOXN1	chr9
A_11_P187243	3.138462	1.6500578	3.138462	up		chr12
A_11_P0000017470	5.068832	2.3416533	5.068832	up		chr34
A_11_P054861	6.2982607	2.6549535	6.2982607	up	DMD	chrX
A_11_P084236	-3.688194	-1.8829145	3.688194	down	KHDRBS3	chr13
A_11_P217073	6.461746	2.691924	6.461746	up		chr13
A_11_P0000033430	-7.5350156	-2.9136105	7.5350156	down	DPT	chr7
A_11_P109626	-3.3287854	-1.7349958	3.3287854	down	LDB2	chr3
A_11_P110831	-3.5910296	-1.8443975	3.5910296	down	AGPAT4	chr1
A_11_P00000687	-4.3470154	-2.1200252	4.3470154	down		chr37

A_11_P080121	5.0892467	2.3474522	5.0892467	up		chr22
A_11_P058156	6.9851484	2.8042908	6.9851484	up		chr37
A_11_P0000021872	-8.182733	-3.0325828	8.182733	down		chr1
A_11_P123086	8.800105	3.1375208	8.800105	up	LAIR1	chr1
A_11_P0000030933	4.1192217	2.0423717	4.1192217	up	ADGRB2	chr2
A_11_P109756	3.061574	1.6142735	3.061574	up	APBB2	chr3
A_11_P0000038322	-4.1714673	-2.060555	4.1714673	down		chr12
A_11_P191488	-33.83882	-5.0806074	33.83882	down		chr6
A_11_P151283	3.0230253	1.595993	3.0230253	up		chr27
A_11_P106551	-4.22082	-2.0775232	4.22082	down		chr5
A_11_P0000023353	-4.007389	-2.0026627	4.007389	down	ABCC11	chr2
A_11_P155013	3.0902278	1.6277132	3.0902278	up		chr1
A_11_P066856	-3.4891474	-1.8028746	3.4891474	down	BMP2	chr24
A_11_P110161	3.0832465	1.6244502	3.0832465	up	CD226	chr1
A_11_P194318	23.890757	4.5783806	23.890757	up		chr37
A_11_P167308	-3.1967263	-1.6765952	3.1967263	down		chr11
A_11_P0000026686	6.6224456	2.727364	6.6224456	up	TMPRSS11D	chr13
A_11_P0000033986	3.4698465	1.7948718	3.4698465	up	IGFBP4	chr9
A_11_P211568	-3.7057922	-1.889782	3.7057922	down		chr20
A_11_P0000024685	3.7915635	1.9227929	3.7915635	up	SERPINE1	chr6
A_11_P186903	-3.8146987	-1.9315691	3.8146987	down	ENO3	chr5
A_11_P187358	-23.246864	-4.5389643	23.246864	down		chr24
A_11_P0000029960	-3.6345623	-1.8617816	3.6345623	down	NGEF	chr25
A_11_P0000022341	-8.9194145	-3.156949	8.9194145	down	BBOX1	chr21
A_11_P128001	-3.4712436	-1.7954526	3.4712436	down	GRHL1	chr17
A_11_P0000034918	-4.002654	-2.000957	4.002654	down	TREX2	chrX
A_11_P0000035125	-3.6298022	-1.8598909	3.6298022	down	ECSCR	chr2
A_11_P0000022889	8.136793	3.0244603	8.136793	up		chr26

A_11_P093731	-4.4897275	-2.166628	4.4897275	down		chr8
A_11_P059266	3.7108138	1.8917356	3.7108138	up	SNCA	chr32
A_11_P161958	3.683745	1.8811731	3.683745	up		chr26
A_11_P0000021493	6.456456	2.6907425	6.456456	up	LAMB1	chr18
A_11_P196068	-5.312906	-2.409501	5.312906	down		chr36
A_11_P0000024462	-3.586534	-1.8425903	3.586534	down		chr5
A_11_P0000024339	-4.908716	-2.2953458	4.908716	down	HSPB3	chr4
A_11_P148078	4.3262477	2.1131163	4.3262477	up	IL13RA2	chrX
A_11_P0000015115	3.5267222	1.8183279	3.5267222	up		chr6
A_11_P0000041401	4.901455	2.29321	4.901455	up		chr30
A_11_P079036	-4.934111	-2.3027902	4.934111	down	LMOD2	chr14
A_11_P148353	-5.236162	-2.3885098	5.236162	down		chr28
A_11_P053206	3.6812205	1.8801842	3.6812205	up	TLE1	chr1
A_11_P207758	-3.146836	-1.653902	3.146836	down	OSBPL1A	chr7
A_11_P186588	9.817267	3.2953215	9.817267	up	LAMB1	chr18
A_11_P195673	4.1476765	2.0523033	4.1476765	up		chr22
A_11_P067516	3.603989	1.8495946	3.603989	up		chr24
A_11_P0000031402	-3.411871	-1.7705631	3.411871	down	ELOVL6	chr32
A_11_P0000019854	4.5426903	2.183547	4.5426903	up	MX2	chr31
A_11_P088281	-8.685096	-3.1185417	8.685096	down	LOC102157231	chr17
A_11_P0000033881	5.676152	2.5049133	5.676152	up	JAG2	chr8
A_11_P0000015620	-4.097101	-2.0346036	4.097101	down	PDLIM3	chr16
A_11_P000008882	3.3629076	1.7497091	3.3629076	up		chr9
A_11_P062161	3.0584903	1.6128197	3.0584903	up	TRIM69	chr30
A_11_P050941	-7.0827346	-2.8243065	7.0827346	down	HSD17B14	chr1
A_11_P156838	3.3401039	1.739893	3.3401039	up		chr19
A_11_P0000038837	-3.518358	-1.8149023	3.518358	down		chr24
A_11_P090656	3.1199489	1.6415224	3.1199489	up	AIF1	chr12

A_11_P0000039495	-3.191569	-1.6742659	3.191569	down		chr37
A_11_P055811	-4.797581	-2.2623072	4.797581	down	NECTIN4	chr38
A_11_P064186	-4.4439425	-2.1518402	4.4439425	down		chr29
A_11_P0000031106	-4.3679214	-2.126947	4.3679214	down	PLA2G4D	chr30
A_11_P139171	-3.5741181	-1.8375874	3.5741181	down	CLDN22	chr16
A_11_P0000030354	5.1238165	2.3572187	5.1238165	up	PKP2	chr27
A_11_P156648	-11.827597	-3.564085	11.827597	down	ACTN2	chr4
A_11_P166378	21.630022	4.434963	21.630022	up		chr15
A_11_P0000034753	-5.231834	-2.3873167	5.231834	down	POF1B	chrX
A_11_P0000033888	3.9093041	1.9669118	3.9093041	up		chr9
A_11_P065126	-6.1887383	-2.6296453	6.1887383	down	SPTSSB	chr34
A_11_P103886	-13.085729	-3.7099223	13.085729	down	ACTN2	chr4
A_11_P0000033597	3.0504813	1.6090369	3.0504813	up	TWSG1	chr7
A_11_P051116	3.1328988	1.6474981	3.1328988	up	CD40	chr24
A_11_P0000033963	5.6278977	2.4925961	5.6278977	up	FKBP10	chr9
A_11_P050496	-9.938045	-3.312962	9.938045	down	ADORA2A	chr26
A_11_P0000016618	-11.777983	-3.5580206	11.777983	down		chr2
A_11_P000003744	-7.718122	-2.9482498	7.718122	down		chr36
A_11_P161778	6.535764	2.708356	6.535764	up	ST3GAL6	chr33
A_11_P076891	-4.6433163	-2.2151556	4.6433163	down	KANK3	chr20
A_11_P0000015997	-4.859219	-2.2807245	4.859219	down	PKDCC	chr17
A_11_P0000020896	6.3669977	2.6706133	6.3669977	up	SULT1D1	chr13
A_11_P133001	3.1322205	1.6471858	3.1322205	up	RASGRP2	chr18
A_11_P000006760	4.474941	2.1618686	4.474941	up		chr31
A_11_P0000029562	8.188923	3.0336738	8.188923	up		chr23
A_11_P0000030832	5.065432	2.3406854	5.065432	up	FGF1	chr2
A_11_P114041	-3.093913	-1.6294327	3.093913	down	CLCN4	chrX
A_11_P104221	5.446436	2.4453125	5.446436	up	MACROH2A2	chr4

A_11_P137386	4.9808946	2.3164048	4.9808946	up	IGFBP4	chr9
A_11_P0000032682	-3.57538	-1.8380966	3.57538	down	ALOX12B	chr5
A_11_P0000030112	16.132904	4.0119343	16.132904	up	CRYBB1	chr26
A_11_P102481	-3.5237672	-1.8171186	3.5237672	down		chr2
A_11_P0000021194	3.2446394	1.6980581	3.2446394	up	RARRES2	chr16
A_11_P0000016794	4.839318	2.2748036	4.839318	up		chr20
A_11_P085581	3.3374875	1.7387624	3.3374875	up	HPDL	chr15
A_11_P0000025913	4.3301744	2.1144252	4.3301744	up		chr10
A_11_P091531	4.7436905	2.2460098	4.7436905	up	LOC610304	chr12
A_11_P140451	-3.2209978	-1.6875076	3.2209978	down	SBSN	chr1
A_11_P208723	-140.54921	-7.1349316	140.54921	down	MB	chr10
A_11_P132361	5.1825395	2.3736591	5.1825395	up		chr10
A_11_P0000019666	3.8780947	1.955348	3.8780947	up	CTSS	chr17
A_11_P0000023801	-5.061103	-2.3394518	5.061103	down	TPRG1	chr34
A_11_P0000033710	-5.3912907	-2.4306307	5.3912907	down	ABHD12B	chr8
A_11_P0000020127	-3.4145832	-1.7717094	3.4145832	down	P2RY1	chr23
A_11_P0000017028	4.966886	2.3123417	4.966886	up		chr2
A_11_P168463	-3.8516612	-1.9454808	3.8516612	down		chr21
A_11_P0000041358	-5.086247	-2.3466015	5.086247	down		chr13
A_11_P066311	-6.11914	-2.613329	6.11914	down	A2ML1	chr27
A_11_P0000017073	65.55726	6.0346837	65.55726	up		chr7
A_11_P090481	-3.4107277	-1.7700796	3.4107277	down	MXD1	chr10
A_11_P0000029955	-3.914869	-1.9689641	3.914869	down	NPPC	chr25
A_11_P0000030457	4.712338	2.236443	4.712338	up	NTF3	chr27
A_11_P110926	-6.273923	-2.6493678	6.273923	down	UNC93A	chr1
A_11_P168558	6.5904346	2.7203736	6.5904346	up	FN1	chr37
A_11_P0000022914	-3.1938663	-1.6753039	3.1938663	down	GTSF1	chr27
A_11_P090941	3.1220894	1.6425118	3.1220894	up	PACSIN1	chr12

A_11_P166843	6.634802	2.7300534	6.634802	up		chr38
A_11_P103091	6.6908474	2.742189	6.6908474	up	TINAGL1	chr2
A_11_P0000021252	6.240585	2.6416812	6.240585	up	DLC1	chr16
A_11_P0000018410	-3.6938913	-1.8851414	3.6938913	down		chr1
A_11_P201458	-299.30695	-8.225482	299.30695	down	MYL1	chr37
A_11_P086176	-15.119488	-3.9183373	15.119488	down	MYBPC1	chr15
A_11_P00000888	3.4421248	1.7832994	3.4421248	up		chr30
A_11_P0000016463	3.1748972	1.6667099	3.1748972	up		chr9
A_11_P190203	3.6099486	1.8519783	3.6099486	up	SLC44A5	chr6
A_11_P0000033235	-5.904423	-2.5617962	5.904423	down		chr6
A_11_P064416	14.219795	3.8298287	14.219795	up	FABP12	chr29
A_11_P206693	-3.9197826	-1.9707737	3.9197826	down	DUSP14	chr9
A_11_P155428	-5.0006757	-2.322123	5.0006757	down		chrX
A_11_P093621	4.5207014	2.1765466	4.5207014	up	GPR65	chr8
A_11_P0000031136	10.330068	3.3687778	10.330068	up	SHC4	chr30
A_11_P0000026955	-17.47119	-4.126906	17.47119	down	SLC5A9	chr15
A_11_P0000028131	-7.6869965	-2.94242	7.6869965	down	SERPINB13	chr1
A_11_P0000023989	-339.1283	-8.405687	339.1283	down	MYL1	chr37
A_11_P127181	-4.4609957	-2.1573658	4.4609957	down		chr9
A_11_P051576	-3.7876456	-1.9213014	3.7876456	down	RBP4	chr28
A_11_P084791	3.7008874	1.8878713	3.7008874	up	LOC611685	chr13
A_11_P076146	4.2815213	2.0981236	4.2815213	up	COMP	chr20
A_11_P0000019696	10.995206	3.4588027	10.995206	up	MAOB	chrX
A_11_P094566	-5.375007	-2.4262667	5.375007	down	MARCHF3	chr11
A_11_P0000040925	-3.6791492	-1.8793721	3.6791492	down		chr1
A_11_P0000032266	3.1864138	1.6719337	3.1864138	up	CABCOCO1	chr4
A_11_P0000039088	6.281374	2.6510801	6.281374	up		chr18
A_11_P114161	3.1001613	1.6323433	3.1001613	up	SCML2	chrX

A_11_P0000017580	9.103305	3.1863904	9.103305	up		chr17
A_11_P055916	-3.4765127	-1.7976408	3.4765127	down	ACKR1	chr38
A_11_P0000030834	3.1963122	1.6764083	3.1963122	up	KCTD16	chr2
A_11_P0000041135	4.975591	2.314868	4.975591	up	NTRK2	chr1
A_11_P192188	4.231847	2.0812874	4.231847	up	SECISBP2L	chr30
A_11_P073181	-3.6443925	-1.8656783	3.6443925	down	CD59	chr18
A_11_P126441	-3.4953885	-1.8054528	3.4953885	down	FLRT3	chr24
A_11_P086446	4.565294	2.1907077	4.565294	up		chr15
A_11_P0000030548	3.105683	1.6349106	3.105683	up	SCD	chr28
A_11_P212593	-4.0162134	-2.005836	4.0162134	down		chr15
A_11_P0000028498	-3.968443	-1.9885731	3.968443	down	SULT2B1	chr1
A_11_P0000018357	4.8276668	2.271326	4.8276668	up		chr1
A_11_P053796	28.459026	4.8308144	28.459026	up	MMP9	chr24
A_11_P200298	4.3158617	2.1096487	4.3158617	up		chr15
A_11_P0000023629	4.0422745	2.0151672	4.0422745	up		chr32
A_11_P0000033974	-153.63127	-7.263328	153.63127	down	KRT33A	chr9
A_11_P0000033719	-4.5176616	-2.1755762	4.5176616	down	BMP4	chr8
A_11_P0000034917	-5.002855	-2.3227515	5.002855	down		chrX
A_11_P00000996	3.9967399	1.9988236	3.9967399	up	SLC12A4	chr5
A_11_P208413	3.5429583	1.8249545	3.5429583	up	TGFBR3	chr6
A_11_P0000025302	6.021927	2.5902252	6.021927	up	FAM20A	chr9
A_11_P200708	-5.2946568	-2.4045372	5.2946568	down		chr11
A_11_P190298	-247.26488	-7.9499135	247.26488	down	LOC609402	chr21
A_11_P054821	3.4075413	1.7687311	3.4075413	up	TACR1	chr17
A_11_P0000020193	3.1118295	1.637763	3.1118295	up	GFI1	chr6
A_11_P054732	8.851905	3.145988	8.851905	up		
A_11_P0000022890	12.112958	3.5984793	12.112958	up		chr26
A_11_P051621	3.0522997	1.6098967	3.0522997	up	ABCC5	chr34

A_11_P058891	-3.7109277	-1.8917799	3.7109277	down		chr33
A_11_P152968	-6.524985	-2.7059746	6.524985	down		chr4
A_11_P098866	3.887729	1.9589276	3.887729	up	NPL	chr7
A_11_P074441	3.6866572	1.8823133	3.6866572	up	RASGRP2	chr18
A_11_P119056	-3.6254044	-1.8581419	3.6254044	down	LOC612422	chr6
A_11_P132936	5.6069064	2.487205	5.6069064	up		chr9
A_11_P148513	-5.3976235	-2.4323244	5.3976235	down	ALDH3A1	chr5
A_11_P0000019145	-4.1916747	-2.0675268	4.1916747	down		chrX
A_11_P105831	-5.2187443	-2.3837028	5.2187443	down		chr5
A_11_P114521	8.1405	3.0251174	8.1405	up	MAOB	chrX
A_11_P136986	3.5889578	1.843565	3.5889578	up	CDH11	chr5
A_11_P122961	-11.54352	-3.5290112	11.54352	down		chr14
A_11_P0000031327	-3.1224651	-1.6426854	3.1224651	down	DNMT3L	chr31
A_11_P158243	-4.6009355	-2.2019272	4.6009355	down	FHL1	chrX
A_11_P0000021121	-3.6248555	-1.8579235	3.6248555	down	MYBPC1	chr15
A_11_P0000019725	-6.7906775	-2.7635555	6.7906775	down	CA6	chr5
A_11_P0000011137	-3.0893333	-1.6272955	3.0893333	down		chr21
A_11_P0000027237	-4.240459	-2.0842204	4.240459	down	RAB11FIP1	chr16
A_11_P0000024341	8.540117	3.094256	8.540117	up		chr4
A_11_P177233	4.528211	2.1789412	4.528211	up		chr37
A_11_P0000014199	6.791351	2.7636986	6.791351	up		chr2
A_11_P054906	3.3290484	1.7351098	3.3290484	up	CXCL8	chr13
A_11_P148318	3.5520394	1.8286476	3.5520394	up	CCDC136	chr14
A_11_P0000017295	-14.917574	-3.898941	14.917574	down	H19	chr18
A_11_P104019	-22.527124	-4.4935913	22.527124	down	ACTA1	chr4
A_11_P181168	5.5744534	2.4788303	5.5744534	up		chr32
A_11_P157558	-90.632195	-6.5019517	90.632195	down	TNNT3	chr18
A_11_P186363	3.9057136	1.9655862	3.9057136	up	FZD7	chr37

A_11_P220888	3.7042882	1.8891964	3.7042882	up	TTC38	chr10
A_11_P0000018892	3.6900363	1.883635	3.6900363	up	LOC606786	chr5
A_11_P0000016348	3.4282823	1.7774858	3.4282823	up		chr4
A_11_P174683	100.469536	6.6506143	100.469536	up	C3	chr20
A_11_P093426	-10.286875	-3.362733	10.286875	down	SYNDIG1L	chr8
A_11_P0000027067	10.299252	3.3644676	10.299252	up	EDNRA	chr15
A_11_P0000028132	-5.0180187	-2.327118	5.0180187	down	SERPINB12	chr1
A_11_P202593	5.060201	2.3391948	5.060201	up	NR1H3	chr18
A_11_P067391	-3.4063702	-1.7682352	3.4063702	down	FAM83C	chr24
A_11_P066321	-11.300471	-3.498311	11.300471	down	MFAP5	chr27
A_11_P0000026065	-8.875864	-3.1498876	8.875864	down		chr10
A_11_P121561	5.108361	2.3528605	5.108361	up	STC1	chr25
A_11_P0000040066	5.4288826	2.4406552	5.4288826	up		
A_11_P0000023525	-5.148334	-2.3641057	5.148334	down	RORA	chr30
A_11_P159703	-3.600021	-1.8480053	3.600021	down		chr24
A_11_P0000021044	9.229483	3.2062497	9.229483	up	SHISAL2A	chr15
A_11_P061306	7.117033	2.831276	7.117033	up	EVA1C	chr31
A_11_P081561	4.7574606	2.2501917	4.7574606	up	TTC25	chr9
A_11_P062401	-4.548476	-2.1853833	4.548476	down	MYZAP	chr30
A_11_P122931	3.3870769	1.7600408	3.3870769	up	FN3K	chrUn_JH373243
A_11_P113736	-5.498684	-2.4590864	5.498684	down	DMKN	chr1
A_11_P0000020593	3.6895692	1.8834524	3.6895692	up	CTNNAL1	chr11
A_11_P000009909	-3.103282	-1.6337948	3.103282	down		chr1
A_11_P129021	4.900953	2.2930622	4.900953	up		chr37
A_11_P140676	-4.3566103	-2.1232061	4.3566103	down	DMKN	chr1
A_11_P0000014560	-3.7959032	-1.9244432	3.7959032	down	EPHX3	chr20
A_11_P000003929	3.5465248	1.826406	3.5465248	up		chr22
A_11_P0000021897	3.960361	1.985632	3.960361	up	PEG3	chr1

A_11_P0000027256	3.3728302	1.7539597	3.3728302	up		chr16
A_11_P0000020189	4.327247	2.1134496	4.327247	up	TACR1	chr17
A_11_P0000017228	-20.242926	-4.339346	20.242926	down		chr6
A_11_P183753	13.6393585	3.7697039	13.6393585	up		chr1
A_11_P0000028128	-12.762327	-3.6738195	12.762327	down	SERPINB7	chr1
A_11_P169513	6.661929	2.73594	6.661929	up		chr2
A_11_P216118	3.0582354	1.6126995	3.0582354	up		chr35
A_11_P173688	7.6929517	2.9435372	7.6929517	up		chr1
A_11_P095631	-8.380045	-3.066958	8.380045	down	SHOC1	chr11
A_11_P0000016691	-10.346941	-3.3711324	10.346941	down		chr6
A_11_P140156	10.172346	3.3465805	10.172346	up	LOC608848	chr38
A_11_P089646	-3.2348256	-1.6936879	3.2348256	down	TIMP3	chr10
A_11_P058556	-3.2588618	-1.7043681	3.2588618	down	IGSF11	chr33
A_11_P201883	4.280214	2.097683	4.280214	up	FRZB	chr36
A_11_P163993	-3.7497802	-1.906806	3.7497802	down	ALPK2	chr1
A_11_P081686	-70.87912	-6.147289	70.87912	down	KRT26	chr9
A_11_P0000019803	-3.112813	-1.6382189	3.112813	down	TGM1	chr8
A_11_P136266	23.405338	4.5487657	23.405338	up		chr21
A_11_P168033	19.03307	4.2504363	19.03307	up		chr27
A_11_P0000024726	4.3758965	2.1295786	4.3758965	up	ZG16	chr6
A_11_P059081	3.7086508	1.8908944	3.7086508	up		chr32
A_11_P0000020023	3.0162566	1.5927591	3.0162566	up	SLC6A6	chr20
A_11_P0000039153	3.5082092	1.8107347	3.5082092	up		chr8
A_11_P0000022217	3.2361057	1.6942587	3.2361057	up	ARRDC5	chr20
A_11_P0000023879	-3.8535635	-1.9461932	3.8535635	down	SCN3A	chr36
A_11_P0000039117	18.813694	4.2337112	18.813694	up	SPEG	chr37
A_11_P194313	-5.442729	-2.4443302	5.442729	down		chr1
A_11_P086306	27.615229	4.787392	27.615229	up	TTC29	chr15

A_11_P0000015488	-3.753598	-1.9082742	3.753598	down	ARHGEF18	chr20
A_11_P0000040016	-5.440326	-2.4436932	5.440326	down		chr2
A_11_P0000041070	5.6276393	2.4925299	5.6276393	up		chr1
A_11_P125081	4.0178413	2.0064206	4.0178413	up	NPR3	chr4
A_11_P156263	-289.87228	-8.179274	289.87228	down	ACTA1	chr4
A_11_P151018	3.7513742	1.9074192	3.7513742	up		chr28
A_11_P070381	-3.314202	-1.7286615	3.314202	down	SCARA5	chr25
A_11_P0000025430	4.4015408	2.1380086	4.4015408	up		chr9
A_11_P125891	-3.4487252	-1.7860632	3.4487252	down	HSPA2	chr8
A_11_P209873	3.4884348	1.8025799	3.4884348	up		chr38
A_11_P0000011241	-4.012635	-2.00455	4.012635	down		chr26
A_11_P0000027526	-7.668102	-2.9388695	7.668102	down	LOC483211	chr17
A_11_P0000026624	-8.645971	-3.1120281	8.645971	down	SLC39A4	chr13
A_11_P182633	3.2116504	1.6833148	3.2116504	up		chr2
A_11_P00000915	45.189877	5.4979277	45.189877	up	NOS2	chr9
A_11_P154078	-5.1173983	-2.3554106	5.1173983	down		chr1
A_11_P167388	3.2271857	1.6902766	3.2271857	up	SLC6A6	chr20
A_11_P0000041779	5.2513065	2.3926764	5.2513065	up		chr15
A_11_P0000041990	3.4577763	1.7898445	3.4577763	up		chr16
A_11_P0000021957	3.340552	1.7400866	3.340552	up	APOE	chr1
A_11_P0000030231	-3.5193326	-1.8153019	3.5193326	down	LIPM	chr26
A_11_P0000039087	3.2153795	1.684989	3.2153795	up		chr26
A_11_P0000024648	-7.80682	-2.964735	7.80682	down	TPPP3	chr5
A_11_P130601	5.298447	2.4055696	5.298447	up		chr27
A_11_P0000028288	4.338971	2.117353	4.338971	up	GNA14	chr1
A_11_P0000018648	3.7404952	1.9032292	3.7404952	up		chr1
A_11_P0000026397	5.084672	2.3461547	5.084672	up	PTK7	chr12
A_11_P0000019749	-5.947863	-2.5723715	5.947863	down	PTGS1	chr9

A_11_P0000021722	7.568547	2.9200163	7.568547	up	ENPP1	chr1
A_11_P0000019699	3.61493	1.8539677	3.61493	up	BHLHE41	chr27
A_11_P133726	3.047595	1.6076713	3.047595	up		chr24
A_11_P0000030336	-3.1856735	-1.6715984	3.1856735	down	ENDOU	chr27
A_11_P0000017174	-4.7199526	-2.2387724	4.7199526	down		chr10
A_11_P000002811	12.568251	3.651712	12.568251	up		chr10
A_11_P210608	3.0069597	1.5883055	3.0069597	up	TNNI3	chr1
A_11_P112196	9.677497	3.274634	9.677497	up	TTYH1	chr1
A_11_P0000022296	-3.9184046	-1.9702663	3.9184046	down		chr21
A_11_P076921	3.232172	1.6925039	3.232172	up	VAV1	chr20
A_11_P051431	4.700381	2.2327776	4.700381	up	FN1	chr37
A_11_P0000027228	12.179591	3.6063938	12.179591	up	PLAT	chr16
A_11_P113241	-4.997082	-2.321086	4.997082	down	CNFN	chr1
A_11_P111771	-4.1179733	-2.0419345	4.1179733	down	BARX1	chr1
A_11_P000002695	-3.6367998	-1.8626695	3.6367998	down	HSPB8	chr26
A_11_P0000030985	-3.072645	-1.6194811	3.072645	down	GRHL3	chr2
A_11_P057871	-251.40695	-7.973881	251.40695	down	MYL1	chr37
A_11_P0000015444	4.639699	2.2140312	4.639699	up		chr11
A_11_P203808	6.956701	2.7984033	6.956701	up	FGF1	chr2
A_11_P087396	-10.881048	-3.4437456	10.881048	down	IL1F10	chr17
A_11_P0000020162	-12.6021595	-3.655599	12.6021595	down	CYP1A2	chr30
A_11_P156993	3.6647854	1.8737288	3.6647854	up	FXYD6	chr5
A_11_P079496	-3.0164628	-1.5928578	3.0164628	down	HOXA7	chr14
A_11_P0000016445	4.3413033	2.1181283	4.3413033	up		chr12
A_11_P0000016545	6.0272555	2.5915012	6.0272555	up	IZUMO4	chr20
A_11_P087386	-4.9502645	-2.3075056	4.9502645	down	IL36B	chr17
A_11_P0000041971	19.595623	4.2924595	19.595623	up	CIDEA	chr7
A_11_P210858	3.8532426	1.946073	3.8532426	up		chr3

A_11_P0000030229	-3.6845753	-1.8814983	3.6845753	down		chr26
A_11_P0000028070	10.208948	3.3517623	10.208948	up	CYP27C1	chr19
A_11_P050456	5.9228473	2.5662909	5.9228473	up	PTHLH	chr27
A_11_P207018	3.254185	1.7022963	3.254185	up		chr24
A_11_P100761	-5.472997	-2.452331	5.472997	down	MUSTN1	chrUn_AAEX03020161
A_11_P086071	-3.8191595	-1.9332552	3.8191595	down		chr15
A_11_P0000016813	14.81303	3.888795	14.81303	up		chr20
A_11_P0000021956	27.441236	4.7782736	27.441236	up	APOC1	chr1
A_11_P138721	3.2911248	1.7185807	3.2911248	up		chr26
A_11_P0000014896	7.863913	2.9752474	7.863913	up	DUSP6	chr15
A_11_P0000022666	-3.2210019	-1.6875095	3.2210019	down	THSD1	chr25
A_11_P0000041077	-5.203304	-2.379428	5.203304	down		chr15
A_11_P176078	7.9882054	2.9978714	7.9882054	up		chr4
A_11_P0000040930	5.303039	2.4068193	5.303039	up		chr3
A_11_P0000035030	3.0084128	1.5890026	3.0084128	up		chr18
A_11_P190393	4.4432287	2.1516085	4.4432287	up		chr2
A_11_P0000029420	5.7589755	2.5258121	5.7589755	up	UGGT2	chr22
A_11_P0000011339	-3.0691726	-1.6178498	3.0691726	down		chr11
A_11_P0000029567	-4.0185065	-2.0066595	4.0185065	down		chr23
A_11_P0000019197	-4.9642243	-2.3115683	4.9642243	down		chr18
A_11_P0000019821	-4.7766323	-2.2559938	4.7766323	down	MSX2	chr4
A_11_P0000025218	-3.330709	-1.7358294	3.330709	down	HSPA2	chr8
A_11_P0000028153	5.6289916	2.4928765	5.6289916	up	MOXD1	chr1
A_11_P0000016840	5.340192	2.4168916	5.340192	up	GPRASP2	chrX
A_11_P0000030842	5.473326	2.4524179	5.473326	up	GZMA	chr2
A_11_P0000017272	3.6409636	1.8643203	3.6409636	up		chr17
A_11_P148528	-4.8779144	-2.2862644	4.8779144	down	NCCRP1	chr1
A_11_P0000033967	-3.2873144	-1.7169094	3.2873144	down	KRT19	chr9

A_11_P0000036218	3.473246	1.7962847	3.473246	up	ST6GALNAC2	chr9
A_11_P0000023168	-6.11928	-2.6133618	6.11928	down		chr29
A_11_P0000013975	-3.280563	-1.7139435	3.280563	down	FAM160A1	chr15
A_11_P0000026847	-3.7759123	-1.9168253	3.7759123	down	GGCT	chr14
A_11_P0000030914	-28.768772	-4.8464317	28.768772	down	PHC2	chr2
A_11_P116391	3.1256588	1.6441603	3.1256588	up		chr11
A_11_P185903	-4.167814	-2.059291	4.167814	down		chr5
A_11_P066181	-145.49193	-7.1847954	145.49193	down		chr27
A_11_P000006038	3.2400978	1.6960373	3.2400978	up		chr23
A_11_P0000032746	-3.722987	-1.8964605	3.722987	down	TACSTD2	chr5
A_11_P050901	3.2062476	1.6808858	3.2062476	up	PPARA	chr10
A_11_P196893	-5.317582	-2.4107704	5.317582	down	ACADVL	chr5
A_11_P0000020841	-3.4260383	-1.7765412	3.4260383	down	KHDRBS3	chr13
A_11_P210488	-6.8030424	-2.76618	6.8030424	down		chr25
A_11_P0000018969	5.8077416	2.5379772	5.8077416	up		chr38
A_11_P123881	5.865991	2.5523748	5.865991	up	MAP1B	chr2
A_11_P223878	-3.6547928	-1.8697896	3.6547928	down	PLPP4	chr28
A_11_P0000039297	4.536686	2.1816387	4.536686	up	FBLIM1	chr2
A_11_P181473	6.8322954	2.7723703	6.8322954	up		chrX
A_11_P095571	3.8346643	1.9391003	3.8346643	up	CTNNAL1	chr11
A_11_P0000024914	-3.3685877	-1.7521439	3.3685877	down	CTH	chr6
A_11_P0000020962	-3.1880224	-1.6726618	3.1880224	down		chr14
A_11_P186293	-4.2943873	-2.1024523	4.2943873	down	BLMH	chr9
A_11_P0000023648	3.1386023	1.6501222	3.1386023	up	SNCA	chr32
A_11_P050331	3.0148144	1.5920691	3.0148144	up	CD8A	chr17
A_11_P197073	19.239258	4.265981	19.239258	up		chr3
A_11_P118791	-6.626047	-2.7281485	6.626047	down		chrX
A_11_P133951	-6.3292894	-2.6620436	6.3292894	down		chrX

A_11_P0000019667	-3.3340573	-1.7372789	3.3340573	down	DSG1	chr7
A_11_P0000014427	3.2701392	1.709352	3.2701392	up		chr29
A_11_P0000021998	-5.4538755	-2.4472818	5.4538755	down	DMKN	chr1
A_11_P0000029047	-7.771383	-2.9581714	7.771383	down		chr20
A_11_P170683	3.340085	1.7398849	3.340085	up	COL12A1	chr12
A_11_P0000020562	4.232268	2.081431	4.232268	up	NPR2	chr11
A_11_P169348	3.379634	1.7568669	3.379634	up	CFAP70	chr4
A_11_P108791	-3.6141224	-1.8536453	3.6141224	down	SERINC5	chr3
A_11_P0000040087	3.3017292	1.7232218	3.3017292	up		chrX
A_11_P0000016953	3.0202768	1.5946808	3.0202768	up		chr14
A_11_P069466	-7.4833198	-2.9036784	7.4833198	down	LYVE1	chr21
A_11_P0000021417	-5.302236	-2.406601	5.302236	down		chr17
A_11_P050776	8.362838	3.0639925	8.362838	up	DMD	chrX
A_11_P0000021302	-3.8943636	-1.9613876	3.8943636	down	ATP6V1C2	chr17
A_11_P0000024992	3.5906188	1.8442326	3.5906188	up	F5	chr7
A_11_P058281	5.8268223	2.5427094	5.8268223	up	ST3GAL6	chr33
A_11_P204043	-3.81515	-1.9317398	3.81515	down	NUAK2	chr38
A_11_P095951	-6.3373003	-2.6638684	6.3373003	down	CLDN4	chr6
A_11_P054956	-4.39761	-2.1367197	4.39761	down	HSPB8	chr26
A_11_P059116	4.00144	2.0005193	4.00144	up	PLAC8A	chr32
A_11_P065455	-7.4443474	-2.8961453	7.4443474	down	KRT71	chr27
A_11_P169253	-3.5365272	-1.8223333	3.5365272	down		chr1
A_11_P0000018737	3.1587594	1.659358	3.1587594	up		chr16
A_11_P0000018739	4.466038	2.1589956	4.466038	up		chr1
A_11_P0000017953	-3.030337	-1.5994782	3.030337	down	PKP1	chr7
A_11_P157453	-168.78548	-7.399047	168.78548	down		chr4
A_11_P193163	-3.3308654	-1.7358971	3.3308654	down		chr17
A_11_P222143	-3.9923737	-1.9972467	3.9923737	down	FETUB	chr34

A_11_P058821	7.1036186	2.8285542	7.1036186	up	SLC51A	chr33
A_11_P057981	6.36965	2.671214	6.36965	up	VIL1	chr37
A_11_P0000033738	13.317205	3.7352195	13.317205	up	SIX1	chr8
A_11_P0000021552	-3.1090312	-1.6364651	3.1090312	down	CD59	chr18
A_11_P105091	3.1724007	1.665575	3.1724007	up	ESM1	chr4
A_11_P154368	-3.6044798	-1.849791	3.6044798	down		chr16
A_11_P141095	-3.1056542	-1.6348972	3.1056542	down	CXXC4	chr32
A_11_P0000022720	-4.858267	-2.2804418	4.858267	down	MFAP3L	chr25
A_11_P0000037206	-5.11232	-2.3539782	5.11232	down		chr35
A_11_P0000027330	-4.6654286	-2.2220097	4.6654286	down	KCNS3	chr17
A_11_P167858	-93.130974	-6.541189	93.130974	down	ACTA1	chr4
A_11_P0000019878	3.1233516	1.643095	3.1233516	up	DHDH	chr1
A_11_P091461	-4.1240754	-2.0440707	4.1240754	down	ADGRF4	chr12
A_11_P137026	-6.530042	-2.7070923	6.530042	down		chr17
A_11_P0000026443	3.9888625	1.9959774	3.9888625	up	EYS	chr12
A_11_P193768	5.8888435	2.5579844	5.8888435	up		chr27
A_11_P102086	4.729094	2.2415638	4.729094	up		chr2
A_11_P093346	4.271249	2.094658	4.271249	up		chr8
A_11_P0000016988	4.643783	2.2153006	4.643783	up		chr1
A_11_P000004762	3.8294334	1.9371309	3.8294334	up	LIPA	chr26
A_11_P180573	4.240341	2.0841804	4.240341	up		chr4
A_11_P000005138	-4.4457145	-2.1524153	4.4457145	down	BLMH	chr9
A_11_P168778	5.2239995	2.3851547	5.2239995	up		chr9
A_11_P0000011183	3.1064355	1.6352601	3.1064355	up		chr27
A_11_P050581	-3.8922071	-1.9605885	3.8922071	down	EDN2	chr15
A_11_P0000015420	4.6644526	2.2217078	4.6644526	up	MSRB1	chr6
A_11_P0000020096	-4.2485886	-2.0869837	4.2485886	down	CCL27	chr11
A_11_P174728	-8.445907	-3.0782523	8.445907	down		chr28

A_11_P0000028408	-4.845385	-2.2766113	4.845385	down	EPS8L1	chr1
A_11_P050876	-4.6673813	-2.2226133	4.6673813	down	AOX4	chr37
A_11_P0000040543	3.7916574	1.9228287	3.7916574	up		chr1
A_11_P206063	-111.497536	-6.800868	111.497536	down		chr10
A_11_P0000023938	-7.0489907	-2.8174167	7.0489907	down	SLC40A1	chr37
A_11_P165918	4.3128657	2.1086469	4.3128657	up		chr9
A_11_P071406	3.1332426	1.6476564	3.1332426	up	NEK10	chr23
A_11_P055886	-3.0773914	-1.6217079	3.0773914	down	VSIG8	chr38
A_11_P0000021321	-5.0636396	-2.3401747	5.0636396	down		chr17
A_11_P138356	-3.2593517	-1.7045851	3.2593517	down		chr17
A_11_P202793	-5.027103	-2.3297272	5.027103	down		chr30
A_11_P059076	4.831985	2.272616	4.831985	up	TMEM150C	chr32
A_11_P183898	3.2549088	1.7026172	3.2549088	up		chr1
A_11_P109071	-3.2884684	-1.7174158	3.2884684	down		chr3
A_11_P0000013965	3.5430696	1.8249998	3.5430696	up		chr3
A_11_P112591	-3.658543	-1.8712692	3.658543	down		chr1
A_11_P090711	-4.976473	-2.3151236	4.976473	down	LY6G6C	chr12
A_11_P00000293	3.3421435	1.7407737	3.3421435	up	NEK10	chr23
A_11_P064131	-3.2796829	-1.7135563	3.2796829	down	SDR16C5	chr29
A_11_P0000020101	3.5381699	1.8230033	3.5381699	up	CCL4	chr9
A_11_P087391	-8.318179	-3.0562677	8.318179	down	IL36RN	chr17
A_11_P078496	-3.46467	-1.7927179	3.46467	down		chr16
A_11_P103881	3.4276183	1.7772064	3.4276183	up	RYR2	chr4
A_11_P162178	5.1976657	2.377864	5.1976657	up		chr36
A_11_P063781	4.476349	2.1623225	4.476349	up	RGS10	chr28
A_11_P112551	-6.637274	-2.7305908	6.637274	down	KLK11	chr1
A_11_P0000016665	-3.3571937	-1.7472558	3.3571937	down		chr36
A_11_P0000017317	4.4592195	2.1567912	4.4592195	up		chrX

A_11_P126586	-13.063823	-3.7075052	13.063823	down		chr22
A_11_P0000034762	-3.2520013	-1.7013278	3.2520013	down	PCDH19	chrX
A_11_P0000016910	41.718517	5.382616	41.718517	up	CHI3L1	chr7
A_11_P126116	-3.1135795	-1.6385741	3.1135795	down		chr9
A_11_P0000014057	-3.6819773	-1.8804808	3.6819773	down		chr13
A_11_P0000029777	3.019129	1.5941324	3.019129	up	ZBP1	chr24
A_11_P119216	3.5316703	1.8203506	3.5316703	up		chr17
A_11_P0000026427	5.2253566	2.3855295	5.2253566	up	LOC610304	chr12
A_11_P190758	-9.049265	-3.1778007	9.049265	down	CRYM	chr6
A_11_P0000032654	3.9948406	1.998138	3.9948406	up	VMO1	chr5
A_11_P095421	3.7557685	1.9091082	3.7557685	up	HEMGN	chr11
A_11_P0000031874	20.686544	4.3706207	20.686544	up	IGFBP2	chr37
A_11_P0000027921	-3.067183	-1.6169143	3.067183	down	RPS6KB2	chr18
A_11_P138456	4.3348327	2.1159763	4.3348327	up		chr13
A_11_P055506	-5.2147183	-2.3825893	5.2147183	down	CFH	chr38
A_11_P098071	-3.4426608	-1.783524	3.4426608	down	KIAA1107	chr6
A_11_P069866	-7.4658732	-2.900311	7.4658732	down	POSTN	chr25
A_11_P154223	7.6402583	2.9336214	7.6402583	up		chr6
A_11_P0000019331	3.491442	1.803823	3.491442	up		chr22
A_11_P0000027853	3.5279953	1.8188486	3.5279953	up	NR1H3	chr18
A_11_P155538	5.4868546	2.4559793	5.4868546	up		chr21
A_11_P088246	-6.731157	-2.7508545	6.731157	down	THEM5	chr17
A_11_P217733	-3.2980602	-1.7216177	3.2980602	down		chr5
A_11_P135131	-3.4145718	-1.7717047	3.4145718	down		chr5
A_11_P0000016284	3.2787876	1.7131624	3.2787876	up		chr29
A_11_P062281	4.634583	2.2124395	4.634583	up	GLDN	chr30
A_11_P0000040246	4.8240557	2.2702465	4.8240557	up		chr10
A_11_P0000017140	-4.3143115	-2.1091304	4.3143115	down		chr25

A_11_P057056	-5.9574666	-2.574699	5.9574666	down	ABCB11	chr36
A_11_P108796	-3.3224936	-1.7322664	3.3224936	down	THBS4	chr3
A_11_P091916	4.8294635	2.271863	4.8294635	up	EPHA7	chr12
A_11_P050501	-10.621507	-3.4089165	10.621507	down	MYOZ1	chr4
A_11_P0000026837	-3.9830592	-1.9938769	3.9830592	down	SKAP2	chr14
A_11_P00000343	6.0698957	2.6016717	6.0698957	up	PPP2R2B	chr2
A_11_P210929	-131.12865	-7.034839	131.12865	down	MYLPF	chr6
A_11_P0000016153	-3.739783	-1.9029546	3.739783	down		chr16
A_11_P107726	-7.000241	-2.8074045	7.000241	down	CA6	chr5
A_11_P0000023284	5.053137	2.3371792	5.053137	up	PPP2R2B	chr2
A_11_P0000028449	-4.2145553	-2.0753803	4.2145553	down	KLK8	chr1
A_11_P164978	19.664474	4.2975197	19.664474	up	KRT13	chr9
A_11_P0000033011	-5.515439	-2.4634757	5.515439	down	TMEM130	chr6
A_11_P0000019683	-3.254244	-1.7023225	3.254244	down	EDN1	chr35
A_11_P093786	6.0742626	2.6027093	6.0742626	up	LOC480425	chr8
A_11_P074591	-3.1601012	-1.6599708	3.1601012	down	SLC22A10	chr18
A_11_P199098	-14.803609	-3.887877	14.803609	down	SLC39A4	chr13
A_11_P211833	-3.6406038	-1.8641777	3.6406038	down		chr23
A_11_P0000030814	6.59609	2.721611	6.59609	up	LOC106557817	chr2
A_11_P0000017190	6.2046113	2.6333408	6.2046113	up		chr23
A_11_P0000017070	-3.430828	-1.7785568	3.430828	down		chr16
A_11_P202683	-5.3478737	-2.4189653	5.3478737	down	GDA	chr1
A_11_P162078	3.8466983	1.9436207	3.8466983	up	PIAS2	chr7
A_11_P055261	4.48553	2.1652784	4.48553	up	SAA1	chr21
A_11_P0000016404	10.498572	3.3921213	10.498572	up	REEP2	chr11
A_11_P051436	6.271947	2.6489134	6.271947	up	FN1	chr37
A_11_P212938	3.4992795	1.8070579	3.4992795	up		chr25
A_11_P0000015943	-3.7455378	-1.9051728	3.7455378	down		chr5

A_11_P071806	-3.19388	-1.6753101	3.19388	down	PLSCR4	chr23
A_11_P141210	3.0469837	1.6073818	3.0469837	up	GTF2IRD1	chr6
A_11_P0000021963	3.276441	1.7121296	3.276441	up	PLAUR	chr1
A_11_P0000032683	-6.5906024	-2.7204103	6.5906024	down	ALOXE3	chr5
A_11_P0000032712	3.11731	1.6403017	3.11731	up		chr5
A_11_P0000026817	-3.0078163	-1.5887165	3.0078163	down	MACC1	chr14
A_11_P0000028752	-3.9681609	-1.9884706	3.9681609	down	PDZRN3	chr20
A_11_P0000026224	-4.049114	-2.0176063	4.049114	down	FRMPD1	chr11
A_11_P081791	-8.573339	-3.0998573	8.573339	down	TCAP	chr9
A_11_P0000017223	11.069402	3.4685054	11.069402	up	LAMC3	chr9
A_11_P0000032046	9.089694	3.1842318	9.089694	up	HAPLN3	chr3
A_11_P063651	19.862312	4.3119617	19.862312	up	ACSL5	chr28
A_11_P051416	-17.768333	-4.1512365	17.768333	down	TTN	chr36
A_11_P0000034640	7.175043	2.8429875	7.175043	up	NDP	chrX
A_11_P098671	-3.7756205	-1.9167137	3.7756205	down	CD55	chr7
A_11_P0000019992	4.3352065	2.1161008	4.3352065	up	ADORA2A	chr26
A_11_P054836	10.447118	3.3850331	10.447118	up	FN1	chr37
A_11_P0000019723	-4.3343515	-2.115816	4.3343515	down	SLC46A2	chr11
A_11_P0000028298	-5.9860506	-2.5816045	5.9860506	down	GDA	chr1
A_11_P000001659	-15.930315	-3.993703	15.930315	down	HOXA3	chr14
A_11_P177918	-3.3504906	-1.7443724	3.3504906	down	TIMP3	chr10
A_11_P0000022200	-3.8916295	-1.9603744	3.8916295	down	KANK3	chr20
A_11_P050186	-5.7478814	-2.5230303	5.7478814	down	SLC6A4	chr9
A_11_P0000027928	-3.761472	-1.9112973	3.761472	down	KDM2A	chr18
A_11_P062276	-4.3889704	-2.1338825	4.3889704	down	TNFAIP8L3	chr30
A_11_P0000032103	-6.01718	-2.5890875	6.01718	down	CPZ	chr3
A_11_P0000039789	-3.0572724	-1.6122451	3.0572724	down		chr17
A_11_P190843	-3.0855596	-1.6255322	3.0855596	down	MYOZ1	chr4

A_11_P085421	-5.071421	-2.34239	5.071421	down	PDZK1IP1	chr15
A_11_P0000023436	18.106182	4.1784105	18.106182	up	FBXO2	chr2
A_11_P0000021444	-3.2088501	-1.6820564	3.2088501	down	ANKRD35	chr17
A_11_P179153	-4.3574386	-2.1234803	4.3574386	down	RGS4	chr38
A_11_P055156	3.8975766	1.9625773	3.8975766	up	SLC12A4	chr5
A_11_P0000028442	3.1218407	1.6423969	3.1218407	up	LOC100686511	chr1
A_11_P0000021537	-4.076855	-2.0274568	4.076855	down	SEMA3C	chr18
A_11_P147853	-4.7595067	-2.250812	4.7595067	down	EDN2	chr15
A_11_P0000015072	-9.513852	-3.2500296	9.513852	down		chr13
A_11_P0000033971	-10.591124	-3.4047837	10.591124	down	KRT32	chr9
A_11_P0000032805	3.2944038	1.7200174	3.2944038	up	ESPN	chr5
A_11_P0000039043	3.3379407	1.7389584	3.3379407	up	RANBP17	chr4
A_11_P134571	117.3354	6.8744946	117.3354	up	STRA6	chr30
A_11_P0000028601	-6.1589847	-2.6226926	6.1589847	down	ACP7	chr1
A_11_P000004807	4.921109	2.2989836	4.921109	up		chr2
A_11_P0000019946	-8.840838	-3.1441832	8.840838	down	DCN	chr15
A_11_P167743	-3.3385444	-1.7392192	3.3385444	down	CD248	chr18
A_11_P087991	6.1239514	2.6144629	6.1239514	up	CD160	chr17
A_11_P0000025562	4.8950763	2.2913313	4.8950763	up		chr21
A_11_P0000037079	3.5204356	1.8157539	3.5204356	up		chr3
A_11_P208458	-3.5530047	-1.8290396	3.5530047	down		chr12
A_11_P0000027554	3.1372912	1.6495194	3.1372912	up	INHBA	chr18
A_11_P000007321	16.40082	4.035696	16.40082	up		chr7
A_11_P148433	4.554138	2.1871781	4.554138	up	TCP11	chr12
A_11_P148608	4.384777	2.1325035	4.384777	up	MPP6	chr14
A_11_P0000024717	-147.04932	-7.200156	147.04932	down	MYLPF	chr6
A_11_P0000025429	7.646839	2.9348636	7.646839	up	CCL23	chr9
A_11_P0000031821	-5.650036	-2.49826	5.650036	down		chr37

A_11_P178153	4.729413	2.241661	4.729413	up		chr8
A_11_P0000026403	4.9741774	2.314458	4.9741774	up	DLK2	chr12
A_11_P0000025274	7.2650723	2.8609772	7.2650723	up	LOC480425	chr8
A_11_P0000018601	3.1393232	1.6504536	3.1393232	up		chr7
A_11_P0000015383	4.7006326	2.2328548	4.7006326	up	ZNF423	chr2
A_11_P0000010601	-4.26473	-2.0924544	4.26473	down		chr1
A_11_P0000019116	3.3294246	1.7352729	3.3294246	up	TMEM98	chr9
A_11_P0000027528	-4.052953	-2.0189734	4.052953	down	CRNN	chr17
A_11_P171413	4.252431	2.0882878	4.252431	up	DPEP1	chr5
A_11_P0000025856	-3.063711	-1.6152802	3.063711	down	RAB3IP	chr10
A_11_P0000016517	-3.3595603	-1.7482724	3.3595603	down		chr7
A_11_P057186	-5.916197	-2.56467	5.916197	down	GPR155	chr36
A_11_P0000025698	-3.4089763	-1.7693386	3.4089763	down	GDPD2	chrX
A_11_P0000021997	-3.413316	-1.771174	3.413316	down	GAPDHS	chr1
A_11_P123561	-3.6834235	-1.8810472	3.6834235	down		chr35
A_11_P0000026943	-3.9526718	-1.9828281	3.9526718	down	GJB5	chr15
A_11_P188463	-256.1205	-8.000679	256.1205	down	ACTA1	chr4
A_11_P119426	-7.7482104	-2.9538631	7.7482104	down	DPT	chr7
A_11_P110326	-4.5148225	-2.1746693	4.5148225	down	RAB27B	chr1
A_11_P115366	5.4463224	2.4452825	5.4463224	up	TCEAL3	chrX
A_11_P157873	-7.982528	-2.9968457	7.982528	down	OTUB2	chr8
A_11_P059526	-3.0917735	-1.6284347	3.0917735	down	FAM241A	chr32
A_11_P187738	-3.8088617	-1.9293599	3.8088617	down		chr16
A_11_P053266	6.2401576	2.6415825	6.2401576	up	DMD	chrX
A_11_P0000039955	3.1472242	1.6540799	3.1472242	up	ADGRB1	chr13
A_11_P0000024763	3.6784024	1.8790793	3.6784024	up		chr6
A_11_P0000029428	-5.0171137	-2.3268576	5.0171137	down	ITGBL1	chr22
A_11_P201108	16.000645	4.000058	16.000645	up	TMEM144	chr15

A_11_P0000030363	3.894078	1.9612818	3.894078	up	ARNTL2	chr27
A_11_P0000033641	3.5335746	1.8211284	3.5335746	up		chr8
A_11_P0000031894	10.220531	3.3533983	10.220531	up	SLC4A3	chr37
A_11_P053366	-247.02104	-7.94849	247.02104	down	LOC609402	chr21
A_11_P218668	-250.0255	-7.9659314	250.0255	down	HBE1	chr21
A_11_P058981	-3.3985307	-1.7649112	3.3985307	down	SHROOM3	chr32
A_11_P072691	3.0983348	1.6314931	3.0983348	up	EGFR	chr18
A_11_P103946	-3.4252174	-1.7761955	3.4252174	down	DISC1	chr4
A_11_P114966	-3.515487	-1.8137245	3.515487	down	GDPD2	chrX
A_11_P225193	4.9737926	2.3143463	4.9737926	up	MEIS1	chr10
A_11_P0000028434	-7.4370995	-2.89474	7.4370995	down	PPP2R1A	chr1
A_11_P0000027175	-3.8289247	-1.9369392	3.8289247	down	ATP6V0A4	chr16
A_11_P0000039507	4.6272545	2.2101564	4.6272545	up		chr18
A_11_P0000039298	8.576072	3.100317	8.576072	up		chr4
A_11_P209323	-5.5694094	-2.4775243	5.5694094	down	RORA	chr30
A_11_P0000017128	5.1946707	2.3770323	5.1946707	up		chr26
A_11_P0000023162	-3.4715316	-1.7955723	3.4715316	down	SDR16C5	chr29
A_11_P128516	-3.2681584	-1.708478	3.2681584	down		chr9
A_11_P205438	-281.38733	-8.136414	281.38733	down	MYL1	chr37
A_11_P0000017310	7.8116183	2.9656215	7.8116183	up		chr6
A_11_P0000019999	-4.6414704	-2.214582	4.6414704	down	TSHR	chr8
A_11_P111461	-3.4498403	-1.7865295	3.4498403	down	ALDH1A1	chr1
A_11_P077596	-3.118755	-1.6409702	3.118755	down	TRPV6	chr16
A_11_P152253	-3.9282753	-1.973896	3.9282753	down	DGAT2	chr21
A_11_P0000034628	3.334883	1.7376361	3.334883	up	CYBB	chrX
A_11_P000005504	-3.1818748	-1.669877	3.1818748	down		chr30
A_11_P0000031676	-4.0803347	-2.0286875	4.0803347	down	RNF144B	chr35
A_11_P103101	5.4095325	2.435504	5.4095325	up	FABP3	chr2

A_11_P195718	3.7022393	1.8883982	3.7022393	up		chr14
A_11_P0000035525	-4.2369833	-2.0830374	4.2369833	down		chr6
A_11_P0000017261	6.3218007	2.6603355	6.3218007	up		chr1
A_11_P160783	-4.5066347	-2.1720505	4.5066347	down	CYP1B1	chr17
A_11_P0000028450	-4.0675454	-2.0241585	4.0675454	down	KLK5	chr1
A_11_P0000017263	10.352643	3.3719273	10.352643	up		chr8
A_11_P071426	7.708783	2.9465032	7.708783	up	RARB	chr23
A_11_P0000019755	-4.151541	-2.053647	4.151541	down	HSPB8	chr26
A_11_P135386	3.9687984	1.9887023	3.9687984	up		chr7
A_11_P0000021542	3.5145807	1.8133526	3.5145807	up		chr18
A_11_P107806	5.022227	2.3283272	5.022227	up	DPEP1	chr5
A_11_P113426	-6.159109	-2.6227217	6.159109	down	NCCRP1	chr1
A_11_P0000013005	-3.1234848	-1.6431565	3.1234848	down		
A_11_P189778	3.0042038	1.5869827	3.0042038	up	CNTLN	chr11
A_11_P155533	6.274269	2.6494474	6.274269	up	DLC1	chr16
A_11_P000005612	3.7406	1.9032698	3.7406	up		chr3
A_11_P138631	-12.740319	-3.6713295	12.740319	down		chr6
A_11_P157393	-3.1368184	-1.649302	3.1368184	down		chr5
A_11_P0000040799	8.981889	3.167019	8.981889	up		chr6
A_11_P0000025328	-4.127303	-2.0451994	4.127303	down	MAPT	chr9
A_11_P112556	-4.6959395	-2.2314138	4.6959395	down	KLK8	chr1
A_11_P0000031134	4.2002816	2.070486	4.2002816	up	MYEF2	chr30
A_11_P0000022534	-3.078669	-1.6223068	3.078669	down	FLRT3	chr24
A_11_P000004969	-3.628646	-1.8594313	3.628646	down		chr8
A_11_P072776	-6.634471	-2.7299814	6.634471	down	SFRP4	chr18
A_11_P131461	4.3697743	2.1275587	4.3697743	up		chr9
A_11_P0000017408	-5.3002667	-2.406065	5.3002667	down		chr1
A_11_P167523	-3.6454115	-1.8660817	3.6454115	down		chr20

A_11_P174508	-3.4125566	-1.770853	3.4125566	down		chr20
A_11_P154413	4.417155	2.1431174	4.417155	up	GLDC	chr11
A_11_P0000030996	13.459533	3.7505565	13.459533	up	C1QC	chr2
A_11_P174363	3.8360105	1.9396067	3.8360105	up		chr2
A_11_P0000013324	-3.2336838	-1.6931787	3.2336838	down		chr20
A_11_P159578	-3.371321	-1.753314	3.371321	down	THSD1	chr25
A_11_P068066	3.1488879	1.6548424	3.1488879	up	CTSZ	chr24
A_11_P172273	4.071099	2.0254183	4.071099	up		chr24
A_11_P107991	4.6502247	2.2173004	4.6502247	up	HSD17B2	chr5
A_11_P0000016300	7.7947254	2.9624982	7.7947254	up		chr10
A_11_P224678	-3.0164678	-1.5928602	3.0164678	down		chr11
